# C1q/TNF‐related protein 4 mediates proliferation and migration of vascular smooth muscle cells during vascular remodelling

**DOI:** 10.1002/ctm2.1261

**Published:** 2023-05-23

**Authors:** Jingying Liu, Xingbo Long, Hexin Li, Qiuxia Yan, Lu Wang, Ziyu Qin, Hong Zhang

**Affiliations:** ^1^ State Key Laboratory of Vascular Homeostasis and Remodeling, The lnstitute of Cardiovascular Sciences, School of Basic Medical Sciences Peking University Health Science Center Beijing China; ^2^ Department of Cardiology Fuwai Hospital Chinese Academy of Medical Sciences & Peking Union Medical College/National Center for Cardiovascular Diseases Beijing China; ^3^ National Center of Gerontology Beijing Hospital Beijing China; ^4^ Biological Sample Management Center Beijing Hospital Beijing China; ^5^ Center for Human Disease Genomics Peking University Health Science Center Beijing China

**Keywords:** C1QTNF4, neointima formation, proliferation and migration, vascular remodelling, vascular smooth muscle cell

## Abstract

**Background:**

Vascular remodelling is an essential pathophysiological state in many circulatory diseases. Abnormal vascular smooth muscle cell (VSMC) behaviour leads to neointimal formation and may eventually results in major adverse cardiovascular events. The C1q/TNF‐related protein (C1QTNF) family is closely associated with cardiovascular disease. Notably, C1QTNF4 has unique two C1q domains. However, the role of C1QTNF4 in vascular diseases remains unclear.

**Methods:**

C1QTNF4 expression was detected in human serum and artery tissues using ELISA and multiplex immunofluorescence (mIF) staining. Scratch assay, transwell assay and confocal microscopy were used to investigate C1QTNF4 effects on VSMC migration. EdU incorporation, MTT assay and cell counting experiment revealed C1QTNF4 effects on VSMC proliferation. C1QTNF4‐transgenic, C1QTNF4^−/−^ and AAV9‐mediated VSMC‐specific C1QTNF4 restoration C1QTNF4^−/‐^ mouse and rat disease models were generated. RNA‐seq, quantitative real‐time PCR, western blot, mIF, proliferation and migration assays were used to investigate the phenotypic characteristics and underlying mechanisms.

**Results:**

Serum C1QTNF4 levels were decreased in patients with arterial stenosis. C1QTNF4 shows colocalisation with VSMC in human renal arteries. In vitro, C1QTNF4 inhibits VSMC proliferation and migration and alters VSMC phenotype. In vivo, an adenovirus‐infected rat balloon injury model, C1QTNF4‐transgenic and C1QTNF4^−/−^ mouse wire‐injury models with or without VSMC‐specific C1QTNF4 restoration were established to mimic the VSMC repair and remodelling. The results show that C1QTNF4 decreases intimal hyperplasia. Especially, we displayed the rescue effect of C1QTNF4 in vascular remodelling using AAV vectors. Next, transcriptome analysis of artery tissue identified the potential mechanism. In vitro and in vivo experiments confirm that C1QTNF4 ameliorates neointimal formation and maintains vascular morphology by downregulating the FAK/PI3K/AKT pathway.

**Conclusions:**

Our study demonstrated that C1QTNF4 is a novel inhibitor of VSMC proliferation and migration that acts by downregulating the FAK/PI3K/AKT pathway, thus protecting blood vessels from abnormal neointima formation. These results provide new insights into promising potent treatments for vascular stenosis diseases.

## Graphical abstract

- C1QTNF4 levels reduce in carotid artery stenosis patients.
- C1QTNF4 is an important regulator of vascular smooth muscle cell proliferation, migration and alters vascular smooth muscle cell phenotype.
- C1QTNF4 treatment can effectively ameliorate vascular remodelling.
- C1QTNF4 negatively regulates the FAK/PI3K/AKT pathway, thus protecting blood vessels from abnormal neointima formation and maintains vascular morphology.

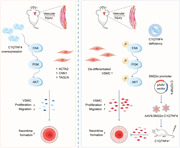

## 1 INTRODUCTION

Vascular remodelling is an active process of structural changes that involves cell proliferation, migration, apoptosis and the production or degradation of the extracellular matrix.^1^ The natural remodelling of arteries is characterised by diffuse intimal thickening (DIT). The thickened intima mainly contains vascular smooth muscle cells (VSMCs), proteoglycans and elastin. DIT is universally present in human atherosclerosis‐prone arteries.^2^ The pathological vascular remodelling is characterised by intimal hyperplasia, which is a healing reaction after mechanical vascular injury. The migration of VSMCs from the media to the intima and abnormal VSMC proliferation are the common cytopathological factors underlying luminal stenosis.^3^ These changes are the main causes of vascular restenosis after percutaneous transluminal coronary angioplasty (PTCA) and late failure of vein grafting. In addition, the accumulation of VSMCs in the intima also occurs in atherosclerosis.^4^ However, the underlying cellular molecular mechanisms of VSMC proliferation and migration are still not fully understood.

VSMCs normally undergo an orderly balance of proliferation and apoptosis. Under pathological conditions, the expression of some cytokines, including platelet‐derived growth factor (PDGF), transforming growth factor β (TGFβ), interleukins and angiotensin II, can affect VSMCs.^5^ These changes trigger the activation of signalling pathways related to proliferation and migration, such as the focal adhesion kinase (FAK)‐Src, PI3K‐AKT, MAPK and JAK‐STAT pathways.^3^ The regulatory network alterations eventually result in uncontrolled hyperplasia and migration of VSMCs and a series of pathological changes in the vascular wall.^6^ The migration of cells begins with the formation of lamellipodia or filopodia at the front of the cell. These protrusions bind the cell to the surface of the substrate beneath it via focal adhesions to make the contents of the cell body flow forwards.^7^ FAK is a highly phosphorylated tyrosine kinase. FAK can be activated by the extracellular matrix or growth factors and phosphorylated at multiple tyrosine sites. Phospho‐FAK (Tyr397) produces SH2‐binding sites for binding to other signalling molecules, such as PI3 kinase p85.^8^ Its kinase activity together with its function as a junction protein and scaffold protein may play an important role in cell migration by promoting the maturation and release of focal adhesions.

The C1q‐ and TNF‐related protein (C1QTNF) family, including C1QTNFs 1 to 15, is a family of adipokines. C1QTNFs are named after their highly conserved C‐terminal complement C1q globular domain. Although C1QTNFs are closely related to adiponectin in sequence and structural organisation, they play unique roles in tissue expression and are closely associated with cardiovascular disease.^9^ The stimulation of C1QTNF1 in macrophages significantly enhances the secretion of proatherosclerotic factors, and oxidised LDL‐induced inflammatory cytokine production is markedly attenuated by treatment with a C1QTNF1 neutralising antibody.^10^ These findings suggest a relationship between C1QTNF1 and atherosclerosis. C1QTNF3 can not only protect against diabetic cardiomyopathy by activating the AMPKα pathway but also protect the heart from cardiac insufficiency, inflammation and cell loss induced by doxorubicin (DOX) via the activation of Sirt1.^11^, ^12^ Thus, C1QTNF3 has therapeutic potential for DOX cardiotoxicity, and its level could be used as a biomarker for coronary artery diseases.^13^ A recent study revealed that C1QTNF9 exerts an anti‐atherosclerotic effect in the early stage by inducing autophagy through the AMPK/mTOR pathway.^14^ Notably, C1QTNF4 is the only C1QTNF family member with two C1q globular domains, which contrasts with other members of the C1QTNF superfamily with only one globular domain.^15^ We first identified C1QTNF4 in 2011 and it has shown the ability to potentiate tumour cell survival and resistance against apoptosis by activating both the NF‐κB and IL6/STAT3 pathways.^16^ C1QTNF4 is mainly expressed in the brain and regulates food intake and energy balance in the hypothalamus.^17^ As for protein structure and function, the full‐length C1QTNF4 protein is composed of 329 amino acids, containing a C1q‐like domain 1 (amino acids 23 to 159) and a C1q‐like domain 2 (amino acids 170 to 314), which were predicted by SMART. C1QTNF4 is the only member with two such domains and may be functionally enhanced. In a subsequent study, we revealed that the two C1q domains of C1QTNF4 significantly attenuate inflammation and glycolipid metabolism in various models.^15^ As for potential interacting proteins, a previous study identified nucleolin as a receptor of C1QTNF4 in monocytes and B cells.^18^ The study suggested that C1QTNF4 interacts with nucleolin via its second C1q‐like domain and nucleolin's C terminus in these cells. Additionally, a recent study has shown that serum C1QTNF4 level may be a potential biomarker for acute coronary syndrome.^19^ However, the role of C1QTNF4 in vascular physiology and disease processes remains unclear.

Considering the positive association between inflammation and neointima formation, we hypothesised that dual C1q domain‐containing C1QTNF4 could be an important regulator of VSMC proliferation/migration, which plays a crucial role in vascular remodelling. That is, we assumed that VSMCs are affected by serum and/or local C1QTNF4 levels when remodelling the vessel wall. To verify this hypothesis, we characterised C1QTNF4 expression and function at the molecular, cellular, and organismal levels. We found that C1QTNF4 attenuates the proliferation and migration of VSMCs and prevents neointima formation after vascular injury. While C1QTNF4 gene knockout significantly exacerbated intimal hyperplasia. Our mechanistic studies clarified that the observed effects were induced, at least in part, by the downregulation of the FAK/PI3K/AKT signalling pathway.

## 2 MATERIALS AND METHODS

Supporting data for this study are available within the article and the online data supplement. Detailed descriptions of materials and experimental procedures are provided in the online data supplement.

### 2.1 Generation of C1QTNF4‐knockout mice and C1QTNF4 transgenic mice

All mice used in this study were of the C57BL/6N background. The C1QTNF4 conventional knockout mouse model was generated by Shanghai Model Organisms Center using CRISPR/Cas9‐mediated genome engineering. The C1QTNF4 gene (NCBI Reference Sequence: NM_026161; Ensembl: ENSMUSG00000040794) is located on mouse chromosome 2. Two exons were identified and exon 2 was selected as target site. Cas9 and gRNA were coinjected into fertilised eggs for knockout mouse production. The pups were genotyped by PCR followed by sequencing analysis. Male and female mice homozygous for a mutation are viable and show normal fertility. The knockout region does not have any other known gene. The C1QTNF4‐TG mouse model was previously published.^20^ C1QTNF4‐TG mice were heterozygotes and expressed human C1QTNF4 protein. All mice were maintained by heterozygous breeding and backcrossed at least six times onto a C57BL/6 background. No obvious abnormalities were observed in these animals. Please see the major resources table in the Supplemental Data. C57BL/6N mice (half male and half female) were placed in a temperature‐controlled environment with a 12‐h light/12‐h dark cycle.

### 2.2 Human sample collection

Serum specimens of healthy Asian subjects or patients with carotid stenosis >70% were included in the study. Detailed descriptions are available in Supplemental Data. Written informed consent was obtained. Human studies received approval by the Institutional Review Board of Beijing Hospital (No.2017BJYYEC‐108‐05) and conformed to the Declaration of Helsinki.

### 2.3 Rat and mouse carotid artery injury models

Sprague‐Dawley rats weighing 350 to 450 g, C1QTNF4^−/−^ mice, sex‐matched littermate control mice, C1QTNF4‐TG mice, and sex‐matched littermate WT mice weighing 19 to 25 g were used in these experiments. General anaesthesia was induced with 100% O_2_/4% isoflurane, and the whole process was maintained with 100% O_2_/2% isoflurane. After the operation, tramadol (10 mg/kg) was injected into the caudal vein of the animal for analgesia. At the end of the experiments, euthanasia was performed using carbon dioxide followed by cervical dislocation. All animal procedures followed protocols approved by the Animal Care and Use Committee at Peking University [approval number LA2014110] and conformed to the guidelines from Directive 2010/63/EU of the European Parliament on the protection of animals used for scientific purposes and the NIH Guide for the Care and Use of Laboratory Animals.

### 2.4 Statistical analysis

Data normality was assessed and confirmed by Shapiro‐Wilk tests. All the results are expressed as the mean ± standard error of the mean (SEM). GraphPad Prism 8.3 software (GraphPad Software, San Diego, CA, USA) was used for statistical analyses. Unpaired two‐tailed Student's *t*‐test was used for comparisons between two groups. If variance was unequal, Welch's *t*‐test was performed. For comparisons between more than two groups, one‐way ANOVA followed by Tukey's posttest or two‐way ANOVA followed by Bonferroni's posttest was performed. *p* values less than .05 were considered statistically significant. Real‐time quantitative PCR data were log transformed for analysis. The number of biological replicates is indicated in individual figures.

## 3 RESULTS

### 3.1 C1QTNF4 levels were lower in patients with arterial stenosis than in those without arterial stenosis

Clinical characteristics of the carotid stenosis and normal carotid groups are shown in Table 1. A total of 30 eligible participants were included. Compared with the normal subjects, patients with carotid stenosis had significantly lower C1QTNF4 levels (Figure S1A). The data suggested that decreased serum C1QTNF4 levels might be related to the prevalence of vascular stenosis disease. Then, we obtained normal renal arteries and renal artery plaques from kidney donors (Table S1). These arteries were stained with haematoxylin & eosin (HE) staining or stained for C1QTNF4 and α‐SMA by immunofluorescence (Figure S1B and C). The expression range of smooth muscle cells was clarified by detecting α‐SMA. C1QTNF4 was mainly expressed in the cytoplasm of α‐SMA positive smooth muscle cell and rarely expressed in the plaque of renal artery, suggesting that C1QTNF4 may be produced by smooth muscle cells (Figure S1D). Taken together, these results suggest that C1QTNF4 reduce during vascular remodelling, and it might be a novel inhibitor of intimal hyperplasia.

**TABLE 1:** Clinical characteristics of participants in carotid artery stenosis and normal carotid groups.

Characteristics	Normal carotid artery (*n* = 12)	Carotid artery stenosis (*n* = 18)	*p* Value
Age (years)	59.3 ± 3.1	58.7 ± 2.0	.862
Male sex (%)	6 (50)	10 (55.6)	>.9999
BMI (kg/m^2^)	24.6 ± 0.7	25.3 ± 0.3	.262
Smoking (%)	5 (41.7)	9 (50)	.722
Heart rate (bpm)	74.3 ± 2.9	73.9 ± 2.3	.923
Total cholesterol (mmol/L)	4.7 ± 0.2	4.9 ± 0.3	.587
LDL cholesterol (mmol/L)	2.7 ± 0.2	3.2 ± 0.1	.045
HDL cholesterol (mmol/L)	1.0 ± 0.1	1.0 ± 0.1	.610
C1QTNF4 (ng/mL)	32.8 ± 2.8	15.7 ± 1.1	<.0001

The measurement data were analysed by the normality test. Values were presented as the mean ± SEM. BMI, body mass index; BP, blood pressure; LDL, low‐density lipoprotein; HDL, high‐density lipoprotein; C1QTNF4, C1q and TNF‐related protein 4.

### 3.2 High expression of C1QTNF4 inhibits the migration of VSMCs

**FIGURE 1:**
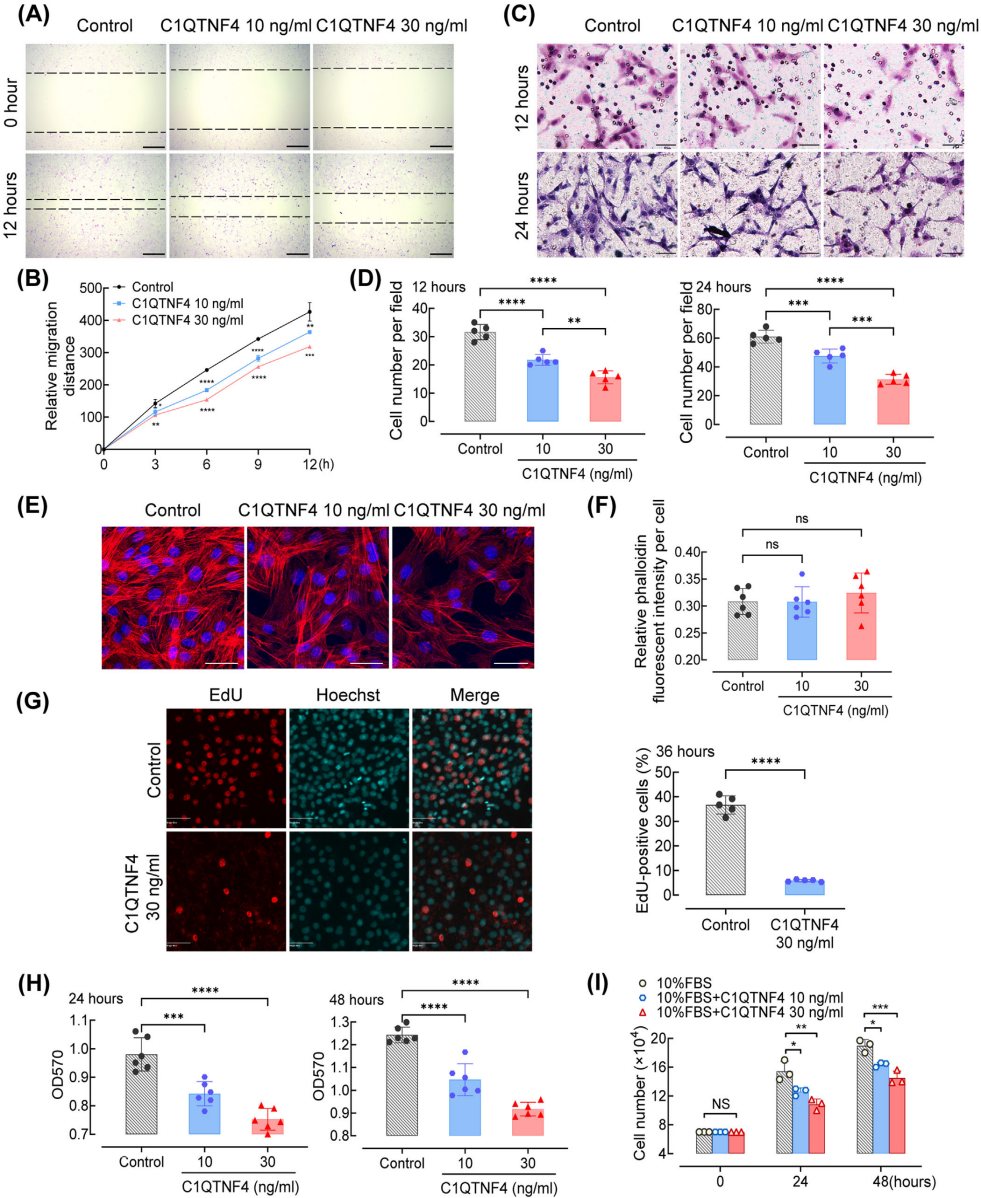
Recombinant C1QTNF4 protein suppresses the migration and proliferation of VSMCs. (A) Confluent VSMC monolayers were treated with or without 10 and 30 ng/mL recombinant C1QTNF4 protein for 24 h before scratch wounding. The cells were kept in culture for an additional 3, 6, 9 and 12 h before analysis. Representative images of the migration assays at 0 h and after 12 h of culture. The dotted line indicates the wound edge. Scale bar, 200 μm. (B) The mean migration distance of VSMCs was quantified at 3, 6, 9 and 12 h after scratching. ± SEM, *n* = 7. (C) VSMCs were seeded on the transwell dishes, exposed to C1QTNF4 10 ng/mL, 30 ng/mL or control for 12 or 24 h, and then subjected to transwell migration assay. Scale bar, 50 μm. (D) The cell number per field was counted in the transwell assay (± SEM; *n* = 5). (E) Representative confocal image of 3 independent experiments showing the effect of recombinant C1QTNF4 protein (10 or 30 ng/mL) on VSMC stress fibres at 48 h. Scale bar, 20 μm. (F) Quantification of relative phalloidin fluorescent intensity per cell with different C1QTNF4 concentrations (± SEM; *n* = 6). (G) Representative images of EdU incorporation in VSMCs incubated with or without 30 ng/mL recombinant C1QTNF4 protein for 36 h. Nuclei were stained with Hoechst 33342 (blue), and proliferating cells were labelled with EdU (red). Scale bars, 50 μm. Percentages of EdU‐positive cells were calculated. ± SEM, *n* = 5 per group. (H) The MTT assay was used to analyse the number of live cells cultured in different concentrations of C1QTNF4 at 24 and 48 h (± SEM; *n* = 6). (I) Cell counting assays were performed with different concentrations of C1QTNF4 (± SEM; *n* = 3). *p < .05, **p < .01, ***p < .001, ****p < .0001; NS indicates no significance.

To examine the influence of C1QTNF4 expression on the migration of VSMCs, we first cultured VSMCs in 20% foetal bovine serum (FBS). Based on the serum C1QTNF4 expression levels in healthy subjects and patients with carotid artery stenosis, recombinant C1QTNF4 protein was added at 10 ng/mL or 30 ng/mL to induce VSMC migration. The migration of VSMCs was observed by cell scratch healing experiments and transwell assays. In the scratch wound‐healing assays, the average migration distance of VSMCs pretreated with recombinant C1QTNF4 protein was significantly shortened, and the trend was dose‐dependent (Figure 1A and B). Consistently, the invasion assays showed that the number of invading cells in the C1QTNF4 group was also markedly lower than that in the control group (Figure 1C and D). In addition, as the dose of C1QTNF4 increased, cell migration decreased. Confocal microscopy showed the same tendency as expected (Figure 1E). Nevertheless, the stress fibre density is not drastically different for the three cases (Figure 1F). Our observations illustrate that C1QTNF4 protein impedes VSMC migration in a dose‐dependent manner.

VSMC phenotypic switch after vascular injury is the key mechanism for stenosis. We treated VSMCs with recombinant C1QTNF4 protein. The VSMC contractile gene expressions were determined by RT‐PCR. Treatment of C1QTNF4 induced the expression of ACTA2(α‐SMA), CNN1(calponin) and TAGLN(SM22α) gene (Figure S2A‐C). Multiple factors, including PDGF, TGFβ and fibroblast growth factor (FGF), can drive VSMC phenotype modulation. We examined whether the expression of C1QTNF4 change under PDGF, TGFβ or FGF stimulation. Relative mRNA levels of C1QTNF4 in VSMCs did not vary with PDGF‐BB, TGFβ or FGF stimulation (Figure S2D‐F). However, C1QTNF4 protein expression increased after PDGF treatment in the culture supernatant, suggesting that C1QTNF4 may be able to act through the supernatant (Figure S2G). Together, these results indicated that C1QTNF4 attenuates contractile VSMCs dedifferentiating into synthetic VSMCs.

### 3.3 C1QTNF4 protein suppresses VSMC proliferation

After observing the relationship between the expression of C1QTNF4 and the migration of VSMCs, we investigated the potential effect of C1QTNF4 on VSMC proliferation. We cultured VSMCs with different concentrations of recombinant C1QTNF4 protein, and the proliferation of VSMCs was monitored by multiple methods. EdU incorporation reflects the activity of DNA synthesis and cell proliferation. C1QTNF4 overexpression significantly inhibited the increase of EdU‐positive cells in VSMCs (Figure 1G). The MTT assay also indirectly showed that the number of viable cells decreased as the C1QTNF4 concentration increased. The cells in the control group maintained the highest viability at both 24‐ and 48‐h incubation times (Figure 1H). We also verified our hypothesis in cell counting experiments (Figure 1I). VSMCs were cultured in 10% FBS, and the number of cells was calculated at 24 or 48 h after culture. The results showed that the number of VSMCs was decreased when involved with C1QTNF4. Together, these results revealed that C1QTNF4 is an inhibitor of VSMC proliferation, and increasing the expression of C1QTNF4 reduced VSMC proliferation activity. The inhibitory effect of C1QTNF4 on VSMCs was time‐ and dose‐dependent. These experiments strongly suggest that the suppressive effect of C1QTNF4 on VSMC proliferation is a general phenomenon.

Besides, apoptosis is a sequential order of cell death that occurs regularly to ensure a homeostatic balance between the rate of cell formation and death. This balance can contribute to cell growth and proliferation. To investigate the function of C1QTNF4 on apoptosis, we treated VSMCs with or without 30 ng/mL recombinant C1QTNF4 protein. Annexin V‐FITC and Propidium Iodide (PI) were detected by immunofluorescence and flow cytometry. The ratio of annexin V‐positive cells and PI‐positive cells are similar in VSMC treated with or without C1QTNF4 protein (Figure S3A‐C). These results indicate that C1QTNF4 was not involved in VSMC apoptosis.

### 3.4 C1QTNF4 ameliorates neointimal hyperplasia after balloon injury in rats

**FIGURE 2:**
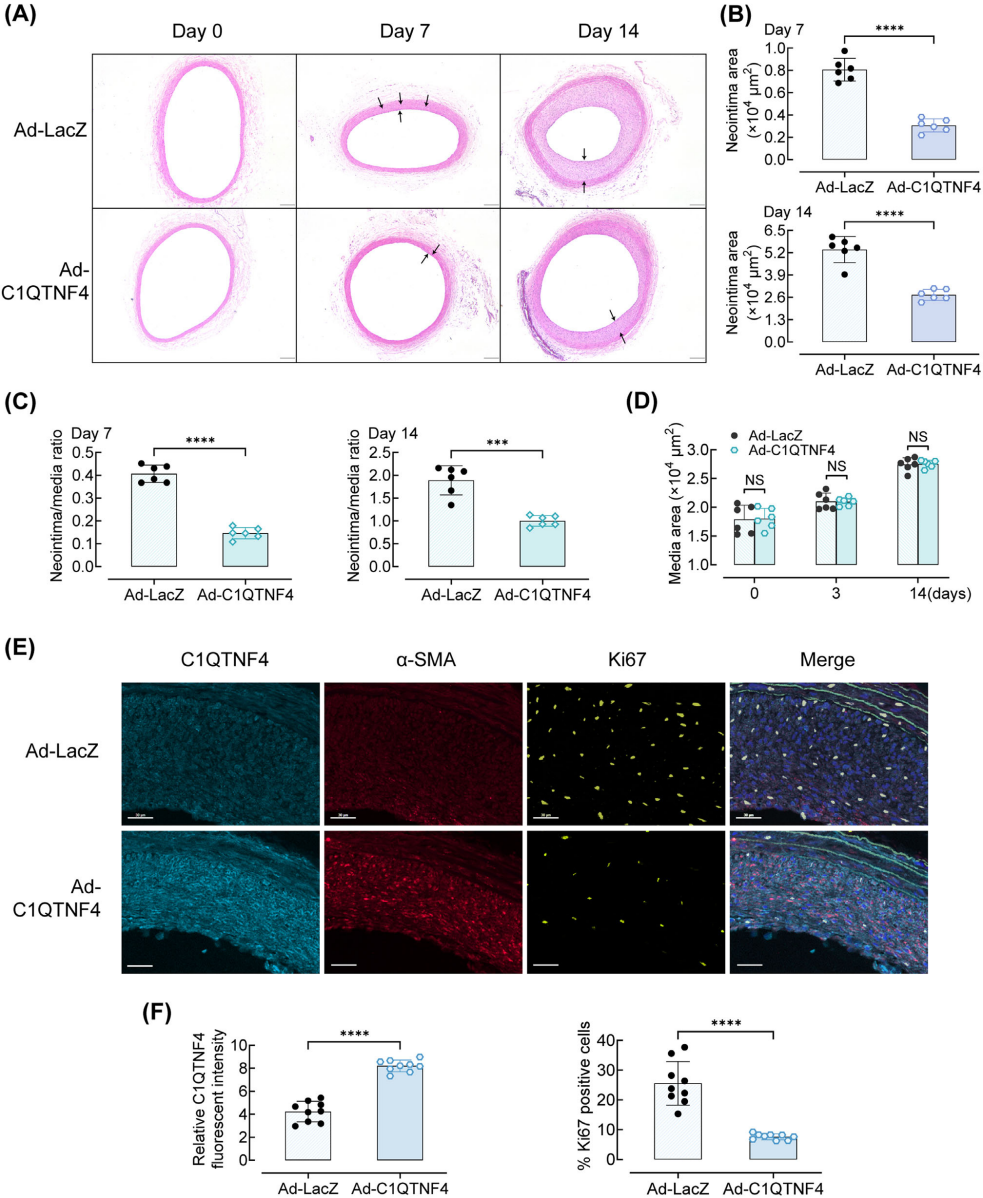
Overexpression of C1QTNF4 inhibits neointima formation in rat common carotid arteries after balloon injury. (A) Representative HE staining of Ad‐C1QTNF4‐infected and Ad‐LacZ‐infected rat common carotid arteries at 0, 7 and 14 days after balloon injury. Scale bar, 200 μm. (B–D) Quantitative analysis of the neointimal area at 7 and 14 days (B), neointima/media ratio at 7 and 14 days (C), and media area (D) (± SEM; *n* = 6 per group). (E) Representative immunofluorescent staining of Ad‐C1QTNF4‐infected and Ad‐LacZ‐infected rat common carotid arteries at 14 days after balloon injury. Slides were simultaneously stained with a multiplex quantitative immunofluorescence (QIF) panel containing C1QTNF4 (cyan), α‐SMA (red), Ki67 (yellow), EL (green) and DAPI (blue). Scale bar, 30 μm. (F) Quantification of the C1QTNF4 expression and Ki67 positive cells in the injured arteries (± SEM, *n* = 9). ***p < .001, ****p < .0001; NS indicates no significance.

Abnormal VSMC proliferation is the biological basis of vascular remodelling, such as neointimal hyperplasia, in response to vascular injury. To further determine the effect of C1QTNF4 on VSMC proliferation in vivo, we used a Sprague‐Dawley rat balloon injury model to examine the progression of neointima formation in common carotid arteries after injury. Ad‐C1QTNF4 or LacZ was intra‐arterially injected to establish the C1QTNF4 overexpression group or control group, as previously described.^21^ Our preliminary results showed that balloon injury leads to transient changes in the expression of C1QTNF4 in the vessel wall. When the intima of carotid arteries is injured, C1QTNF4 accumulates in large amounts locally (Figure S4A). Next, at 0, 7 and 14 days after model establishment, injured right common carotid artery intima samples were taken for sectioning, and changes in intimal hyperplasia were observed. HE staining showed that intimal hyperplasia developed more obviously in the Ad‐LacZ‐infected vessels than in the Ad‐C1QTNF4‐infected vessels after balloon injury (Figure 2A). At 7 days post balloon injury, the neointimal area of the carotid arteries infected with Ad‐LacZ was approximately 3.0‐fold greater than that of arteries treated with Ad‐C1QTNF4 (Ad‐LacZ vs. Ad‐C1QTNF4: 0.83 × 10^4^ ± 0.11 × 10^4^ vs. 0.32 × 10^4^ ± 0.06  × 10^4^ μm^2^; *n* = 6 per group; *p* < .0001; Figure 2B). At 14 days after injury, neointima formation was significantly suppressed and also showed a large gap in the Ad‐C1QTNF4‐infected arteries compared with the control arteries (Ad‐LacZ vs. Ad‐C1QTNF4: 5.67 × 10^4^ ± 0.37 × 10^4^ vs. 2.83 × 10^4^ ± 0.25 × 10^4^ μm^2^; *n* = 6 per group; *p* < .01; Figure 2B). The neointima to media ratio trend was similar to that of the neointimal area in which the ratio of the Ad‐C1QTNF4‐infected artery group was significantly reduced at both day 7 and day 14 (Figure 2C). However, there was no significant difference in the medial area of the balloon‐injured vessels (Figure 2D). It was obvious that Ad‐LacZ‐infected injured arteries had more severe neointimal hyperplasia than Ad‐C1QTNF4‐infected arteries. The tunica media remained almost unchanged.

Moreover, immunofluorescent staining of Ad‐C1QTNF4‐infected and Ad‐LacZ‐infected rat common carotid arteries were performed at 14 days post balloon injury. Slides were simultaneously stained with a multiplex quantitative immunofluorescence (QIF). Relative C1QTNF4 fluorescent intensity in Ad‐C1QTNF4‐infected rat common carotid arteries are respectively 1.9‐folds (*p* < .0001) increased than that in Ad‐LacZ‐infected rat common carotid arteries. In contrast, staining for the proliferation marker Ki67 is significantly decreased in Ad‐C1QTNF4‐infected rat common carotid arteries (Figure 2E and F). To further confirm what cell types in the blood vessel wall that C1QTNF4 overexpression is achieved, we also detected the expression of CD3, CD68 and CD31 in rat common carotid arteries by multiplex QIF. The results suggested that there was no significant difference between Ad‐LacZ group and Ad‐C1QTNF4 group on leukocyte subsets or re‐endothelialisation in rat common carotid arteries (Figure S4B). In addition, since VSMC apoptosis was involved in neointimal formation, we detected apoptosis marker (caspases‐3) as well. No significant influence of C1QTNF4 on apoptosis was observed between the two groups (Figure S4B). Thus, through adenoviral transduction, we indicated that C1QTNF4 is mainly expressed in VSMC and ameliorates neointima formation in balloon‐injured rat carotid arteries.

### 3.5 Inhibition of neointimal formation induced by carotid guide wire injury in C1QTNF4 transgenic mice

**FIGURE 3:**
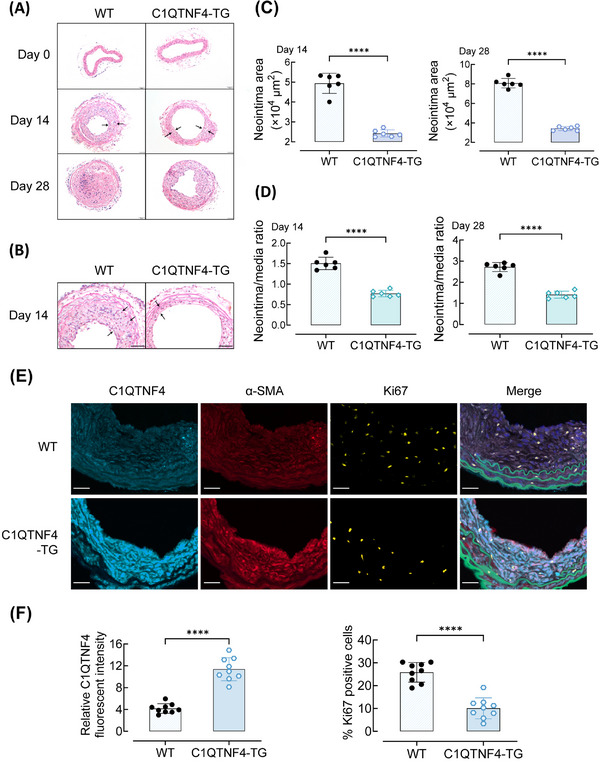
C1QTNF4 reduces wire injury–induced neointima formation in transgenic mouse carotid arteries. (A, B) Representative HE staining of 8‐week‐old C1QTNF4 transgenic (C1QTNF4‐TG) and sex‐matched wild‐type (WT) mouse common carotid arteries on the 0th, 14th and 28th days after wire injury. Scale bar, 50 μm. (C, D) Quantitative analysis of the neointimal area on the 14th and 28th days (C), and the neointima/media ratio on the 14th and 28th days (D) (± SEM; *n* = 6 per group). (E) Representative immunofluorescent staining of WT and C1QTNF4‐TG mouse common carotid arteries at 14 days after wire injury. Slides were simultaneously stained with a multiplex QIF panel containing C1QTNF4 (cyan), α‐SMA (red), Ki67 (yellow), EL (green) and DAPI (blue). Scale bar, 30 μm. (F) Quantification of the expression of C1QTNF4 and Ki67 positive cells in the injured arteries (± SEM, *n* = 9). ****p < .0001.

To further confirm our hypothesis that C1QTNF4 acts as a negative regulator of cell migration and proliferation in vivo, we used a transgenic mouse carotid guide wire‐injury model to mimic the process of VSMC repair and remodelling. The C1QTNF4 transgenic (C1QTNF4‐TG) mouse model was previously published.^20^ The expression of C1QTNF4 was increased in the C1QTNF4‐TG mouse model (Figure S4C and D). Photomicrographs of HE‐stained carotid arteries showed mild‐to‐moderate intimal hyperplasia developing in wire‐injured mouse carotid arteries at 14 days post injury, and intimal hyperplasia became more severe over time. At up to 28 days after surgery, the carotid artery intima of control mice was almost completely occluded, while the neointimal proliferation and stenosis in the C1QTNF4‐TG mice were much milder (Figure 3A and B). Consistent with this observation, the neointimal area of C1QTNF4‐TG mice was substantially decreased compared with that of the control mice (Figure 3C). At 28 days after injury, the neointima/media ratio of the C1QTNF4‐TG group arteries was approximately 51.9% of that of arteries of the control group (Figure 3D). Further, multiplex QIF of WT and C1QTNF4‐TG mouse common carotid arteries were performed at 14 days after wire injury. Both relative C1QTNF4 and α‐SMA fluorescent intensity are significantly increased in C1QTNF4‐TG mouse common carotid arteries. Whereas, Ki67‐positive cells are significantly decreased in C1QTNF4‐TG groups (Figure 3E and F). These results indicated that neointimal formation induced by carotid guide wire injury was inhibited in C1QTNF4‐TG mice, and C1QTNF4 plays roles in VSMCs. In addition, since C1QTNF superfamily is a family of adipokines, we analysed metabolism to exclude potential confounding factors associated with vascular remodelling. No significant changes in weight gain and lipid profile was found between WT and C1QTNF4‐TG mice (Figure S4E and F).

### 3.6 C1QTNF4 deficiency exacerbates neointimal thickening

**FIGURE 4:**
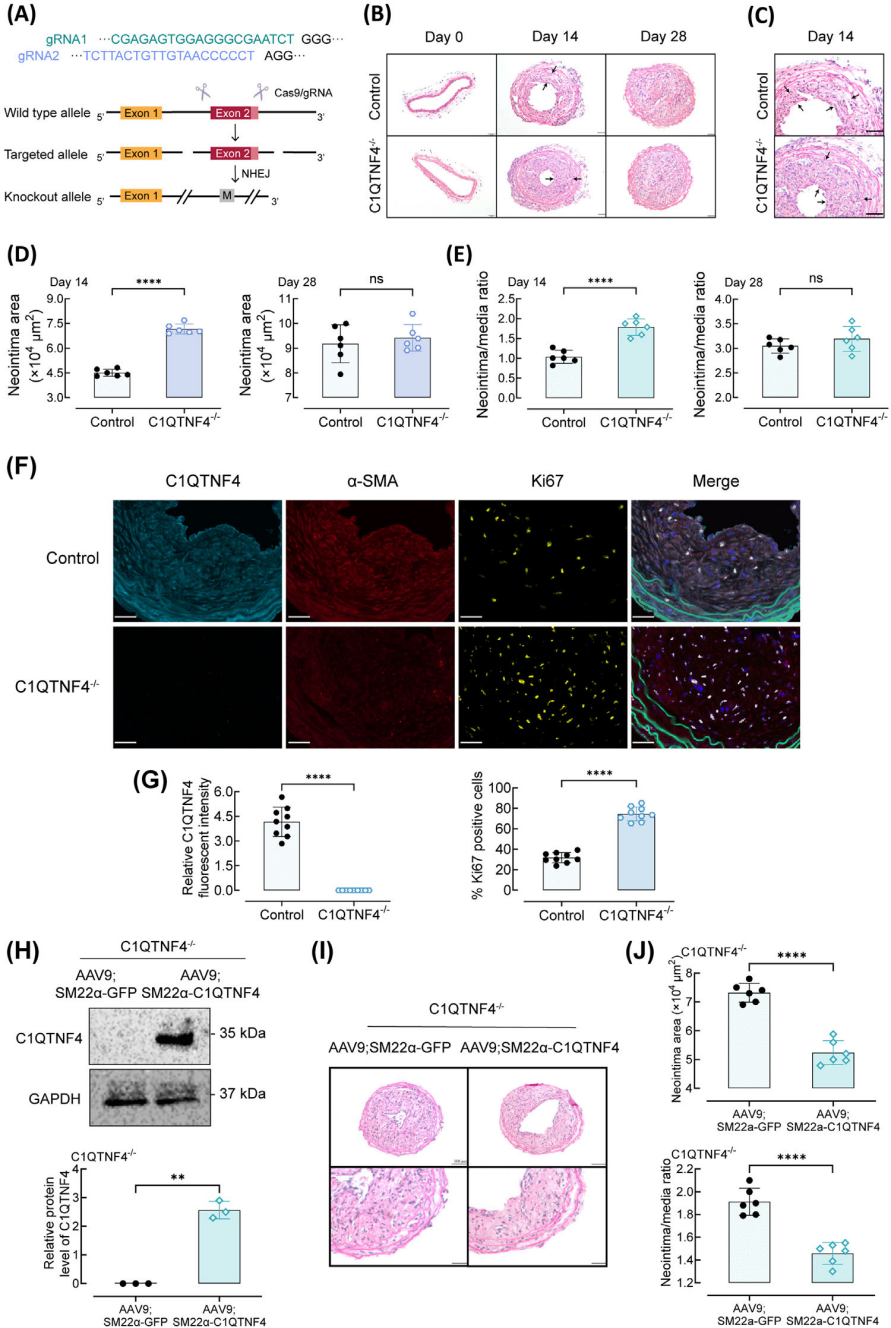
C1QTNF4‐knockout exacerbates wire injury–induced neointimal thickening in mouse carotid arteries and restored C1QTNF4 expression in VSMC exerts a rescue effect. (A) Schematic of the genome engineering targeting strategy of the CRISPR/Cas9‐mediated C1QTNF4 conventional knockout (C1QTNF4^−/−^) mouse model. Exon 2 was selected as target site. NHEJ, non‐homologous end joining. M, mutation. (B, C) Representative HE staining of 8‐week‐old C1QTNF4^−/−^ and sex‐matched littermate control mouse common carotid arteries on the 0th, 14th and 28th days after wire injury. Scale bar, 50 μm. (D, E) Quantitative analysis of the neointimal area on the 14th and 28th days (D), and the neointima/media ratio on the 14th and 28th days (E) (± SEM; *n* = 6 per group). (F) Representative immunofluorescent staining of control and C1QTNF4^−/−^ mouse common carotid arteries at 14 days after wire injury. Slides were simultaneously stained with a multiplex QIF panel containing C1QTNF4 (cyan), α‐SMA (red), Ki67 (yellow), EL (green) and DAPI (blue). Scale bar, 30 μm. (G) Quantification of the C1QTNF4 expression and Ki67 positive cells in the injured arteries (± SEM, *n* = 9). (H) 6‐week‐old C1QTNF4^−/−^ mouse infected with AAV9; SM22α‐C1QTNF4 vectors and sex‐matched C1QTNF4^−/−^ mouse infected with AAV9; SM22α‐GFP vectors. Western blot of C1QTNF4 in the common carotid arteries VSMCs of C1QTNF4^−/−^; AAV9; SM22α‐GFP mice and C1QTNF4^−/−^; AAV9; SM22α‐C1QTNF4 mice. Protein levels of C1QTNF4 were assessed by Western blot analysis (± SEM, *n* = 3). (I) Representative HE staining of C1QTNF4^−/−^; AAV9; SM22α‐C1QTNF4 and C1QTNF4^−/−^; AAV9; SM22α‐GFP mouse common carotid arteries on the 14th day after wire injury. Scale bar, 100 μm, 50 μm. (J) Quantitative analysis of the neointimal area and neointima/media ratio on the 14th day. ± SEM, *n* = 6 per group. **p < .01, ****p < .0001; NS indicates no significance.

We next examined the contribution of C1QTNF4 deletion to VSMC proliferation in vivo to better illustrate the C1QTNF4 effect (Figures 4A and S5A). Under normal conditions, compared with the control group, the C1QTNF4‐knockout group exhibited no difference in the body weight and systemic arterial pressure, indicating that the ablation of C1QTNF4 had no apparent influence on circulation development and function. In C1QTNF4‐knockout mice, carotid guide wire injury resulted in a substantial enlargement of the neointimal lesions at 14 days post injury, which evidently reduced the lumen of the vessel (Figure 4B and C). In contrast, control mice exhibited less stenosis than C1QTNF4‐knockout mice at 14 days post injury. At up to 28 days after surgery, the vessels of both groups were almost completely occluded (Figure 4D and E). In addition, we also performed multiplex QIF to test the expression of C1QTNF4 and Ki67 on control and C1QTNF4‐knockout mouse common carotid arteries. Relative C1QTNF4 fluorescent intensity in C1QTNF4‐knockout mouse was undetected. While, the proliferation marker (Ki67) is obviously increased in C1QTNF4‐knockout mouse common carotid arteries (Figure 4F and G). We also analysed weight gain and lipid profile in control mice and C1QTNF4‐knockout mice. Consistently, body weight, serum levels of total cholesterol and triglyceride were similar between the two groups (Figure S5B and C). Thus, our findings suggest that C1QTNF4 knockout exacerbates neointimal thickening speed in response to injury—C1QTNF4 protects against neointima formation.

Furthermore, we conducted a novel adeno‐associated viral (AAV) vector to further assess the specific role of C1QTNF4. We used SM22α to ensure specific expression in smooth muscle cells. The C1QTNF4 conventional knockout mouse was infected with *AAV9; SM22α‐C1QTNF4* vectors. Thereby, in C1QTNF4‐knockout mice, C1QTNF4 expression was restored in VSMC, and VSMC could secrete C1QTNF4 (Figures 4H and S5D). Our results show that restored C1QTNF4 expression in VSMC only partially ameliorates neointimal formation (Figure 4I and J). This finding implied that VSMCs may affect by both serum and local C1QTNF4 levels when remodelling the vessel wall.

### 3.7 C1QTNF4 regulates VSMC proliferation and migration through the FAK/PI3K/AKT pathway

**FIGURE 5:**
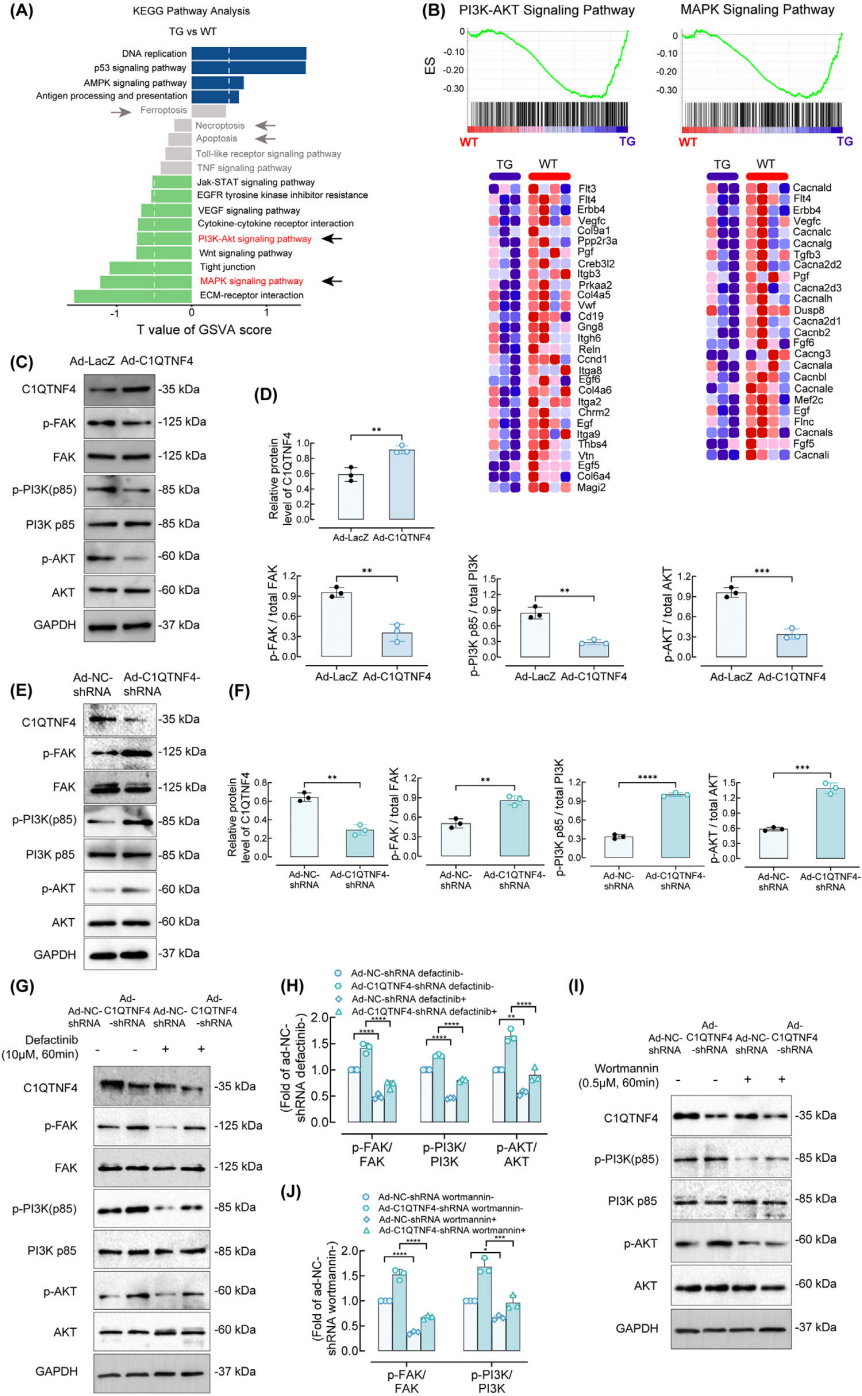
C1QTNF4 regulates vascular VSMC proliferation and migration through the FAK/PI3K/AKT pathway. (A) KEGG pathway GSVA enrichment analysis shows the differences in pathway activities between C1QTNF4‐TG and WT mice common carotid arteries samples collected at 14 days after wire injury. Green bar on the left represented the significantly enriched pathways in WT samples. Blue bar on the right represented the significantly enriched pathways in C1QTNF4‐TG samples. Grey bar represented the not significantly enriched pathways in both WT and C1QTNF4‐TG samples. (B) Enrichment of PI3K‐AKT and MAPK signalling pathways analysed by GSEA. The heatmap below show the represented PI3K‐AKT or MAPK signalling pathways genes between WT and C1QTNF4‐TG groups. The heatmap was generated by the gsea‐3.0.jar software. (C, D) Western blot of VSMCs infected with Ad‐C1QTNF4 or Ad‐LacZ for p‐FAK (Tyr397), FAK, p‐PI3K p85, PI3K p85, p‐AKT (Ser473), AKT and GAPDH as the loading control (C). After culture for 14 days, the relative protein levels in Ad‐C1QTNF4‐infected cells and Ad‐LacZ‐infected cells were assessed by Western blot analysis (± SEM, *n* = 3) (D). (E, F) Likewise, immunoblots of cells infected with Ad‐NC‐shRNA or Ad‐C1QTNF4‐shRNA for p‐FAK, FAK, p‐PI3K p85, PI3K p85, p‐AKT, AKT and GAPDH as the loading control (E). Relative protein levels were assessed (± SEM, *n* = 3) (F). (G, H) VSMCs affected by Ad‐NC‐shRNA or Ad‐C1QTNF4‐shRNA were treated with or without the indicated FAK inhibitors (defactinib at 10 μM) for 1 h, and cell lysates were prepared for immunoblotting for the indicated proteins. Representative western blots are shown. The p‐FAK/t‐FAK, p‐PI3K p85/t‐PI3K p85 and p‐AKT/t‐AKT ratios were calculated. ± SEM, *n* = 3. (I, J) VSMCs infected with Ad‐NC‐shRNA or Ad‐C1QTNF4‐shRNA were treated with or without 0.5 μM wortmannin. Cell lysates were collected and examined by Western blot analysis (± SEM, *n* = 3). **p < .01, ***p < .001, ****p < .0001.

We performed RNA sequencing to investigate the potential underlying mechanism. As shown by the results of KEGG pathway enrichment analysis, there were alterations in the expression of PI3K/AKT and MAPK signalling pathways proteins (Figure 5A). And gene set enrichment analysis further confirmed that the differential genes between C1QTNF4‐TG and WT mice common carotid arteries samples significantly affected PI3K/AKT and MAPK signalling pathway (Figure 5B). The PI3K/AKT and MAPK signalling pathways are classical pathways for smooth muscle cell proliferation and migration during carotid artery injury. We used western blot to verify the potential pathway at the cellular level and found that C1QTNF4 acts through the PI3K/AKT pathway. While the phosphorylation of the MAPK pathways remains unchanged (Figure S6A). We next explored the upstream of PI3K. Previous studies have demonstrated that FAK plays an important role in cell proliferation and migration by promoting the maturation and release of focal adhesions.^22^, ^23^ Since a large body of literature has shown that phosphorylated FAK can activate downstream PI3K‐AKT,^23^, ^24^, ^25^ we wanted to investigate in deep whether the C1QTNF4‐guided regulation of VSMC proliferation and migration is mediated by the FAK/PI3K/AKT pathway. We transfected VSMCs with a C1QTNF4 overexpression adenovirus (Ad‐C1QTNF4) or Ad‐C1QTNF4‐shRNA to observe the effect of C1QTNF4 on the above pathways. There was a significant increase in C1QTNF4 protein in the Ad‐C1QTNF4 group and an obvious reduction in C1QTNF4 in the Ad‐C1QTNF4‐shRNA group compared with the control groups. We next tested whether C1QTNF4 modulates the activity of FAK. The p‐FAK/total FAK ratio in Ad‐LacZ‐infected VSMCs was higher than that in Ad‐C1QTNF4‐infected cells. The ratio in Ad‐NC‐shRNA‐infected cells was lower than that of cells transfected with Ad‐C1QTNF4‐shRNA. These results suggest that C1QTNF4 is an inhibitor of FAK activation. Then, we used western blotting to investigate the expression levels of p‐PI3K p85. The ratio of p‐PI3K p85 to total PI3K p85 was significantly reduced in the Ad‐C1QTNF4‐infected VSMCs on the 14th day. Accordingly, p‐PI3K p85 was increased in Ad‐C1QTNF4‐shRNA‐infected cells compared with Ad‐NC‐shRNA‐infected cells. Finally, the phosphorylation level of AKT was detected to evaluate the activity of AKT. The greyscale density of phosphorylated AKT in the C1QTNF4‐overexpression group was lower than that in the Ad‐LacZ group. Consistently, C1QTNF4 silencing largely reduced the inhibition of AKT activation (Figure 5C‐F). In addition, the phosphorylation of downstream PI3K and AKT was significantly inhibited by the FAK inhibitor defactinib in both the experimental group and the control group (Figure 5G and H). However, the addition of the PI3K inhibitor wortmannin downregulated AKT phosphorylation, while the upstream trend was consistent with the hypothesis mentioned above (Figure 5I and J). Moreover, to establish dependency shRNA‐based knockdown or rescue of the FAK/PI3K/AKT pathways, we also utilised defactinib and wortmannin in C1QTNF4 deficient VSMCs (Figure S7A‐D). Consistently, the addition of the FAK and PI3K inhibitor suppress VSMCs proliferation and migration. Taken together, these data demonstrate the causal relationship that adenovirus‐mediated upregulation of C1QTNF4 suppresses the FAK/PI3K/AKT pathway.

### 3.8 The effect of C1QTNF4 on intima is mediated by FAK/PI3K/AKT in vivo

**FIGURE 6:**
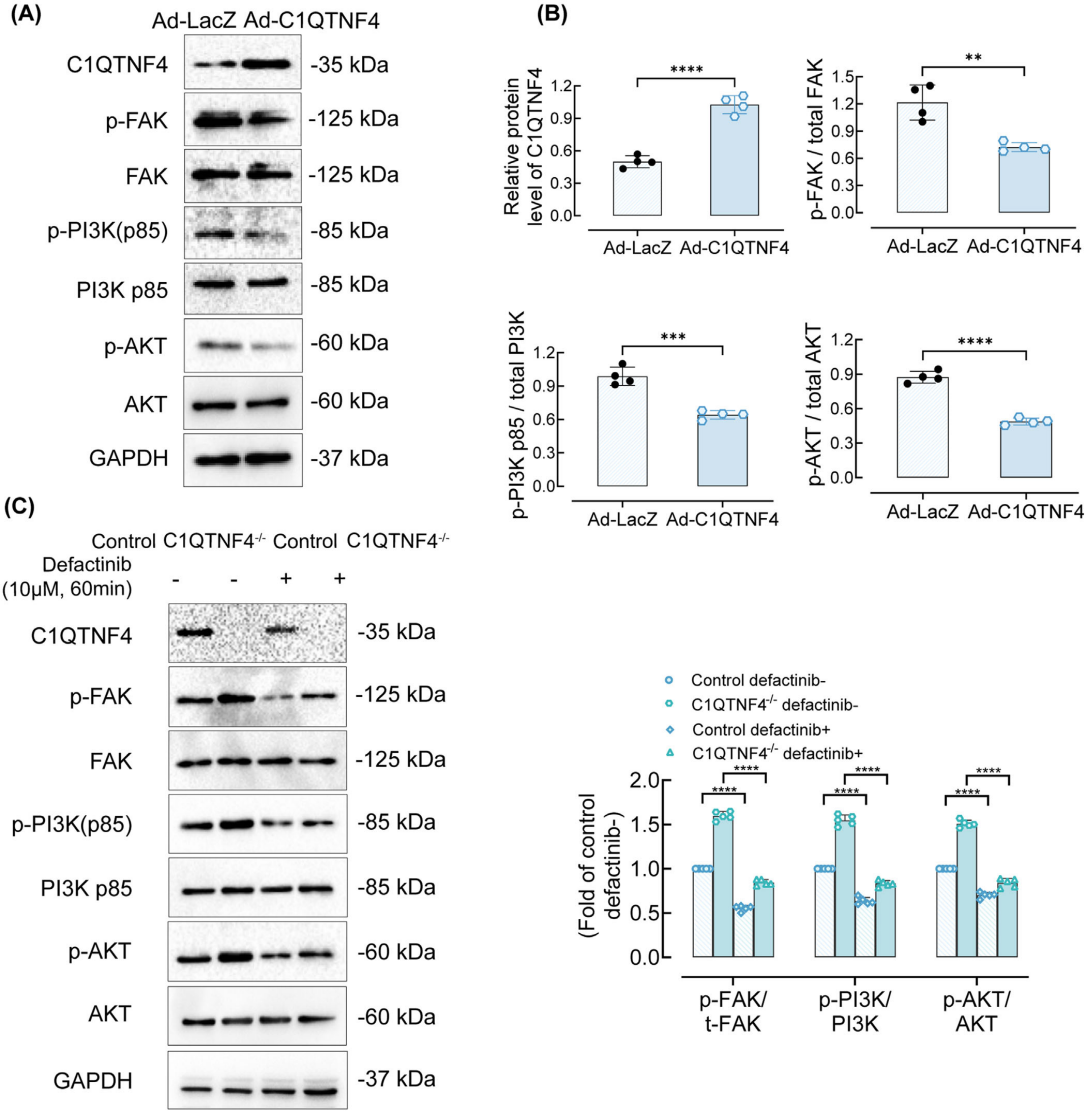
The effect of C1QTNF4 on neointima formation is mediated by the FAK/PI3K/AKT pathway. (A) Rats carotid arteries were temporarily injected with Ad‐C1QTNF4 or Ad‐LacZ for 15 min after balloon injury. After 14 days, the protein was extracted for immunoblotting. Immunoblots of neointima cells infected with Ad‐C1QTNF4 or Ad‐LacZ for C1QTNF4, p‐FAK (Tyr397), FAK, p‐PI3K p85, PI3K p85, p‐AKT (Ser473), AKT, and GAPDH as the loading control. (B) The protein levels of C1QTNF4, the p‐FAK/total FAK ratio, the p‐PI3K p85/total PI3K p85 ratio and the p‐AKT/total AKT ratio were assessed by Western blot analysis (± SEM, *n* = 4). (C) On the 14th day after the establishment of the mice wire‐injury model, some control or C1QTNF4^−/−^ mice carotid arteries tissues were treated with or without defactinib at 10 μM. Representative western blots are shown and the p‐FAK/t‐FAK, p‐PI3K p85/t‐PI3K p85 and p‐AKT/t‐AKT ratios were assessed (± SEM, *n* = 5). **p < .01, ***p < .001, ***p < .0001.

**FIGURE 7:**
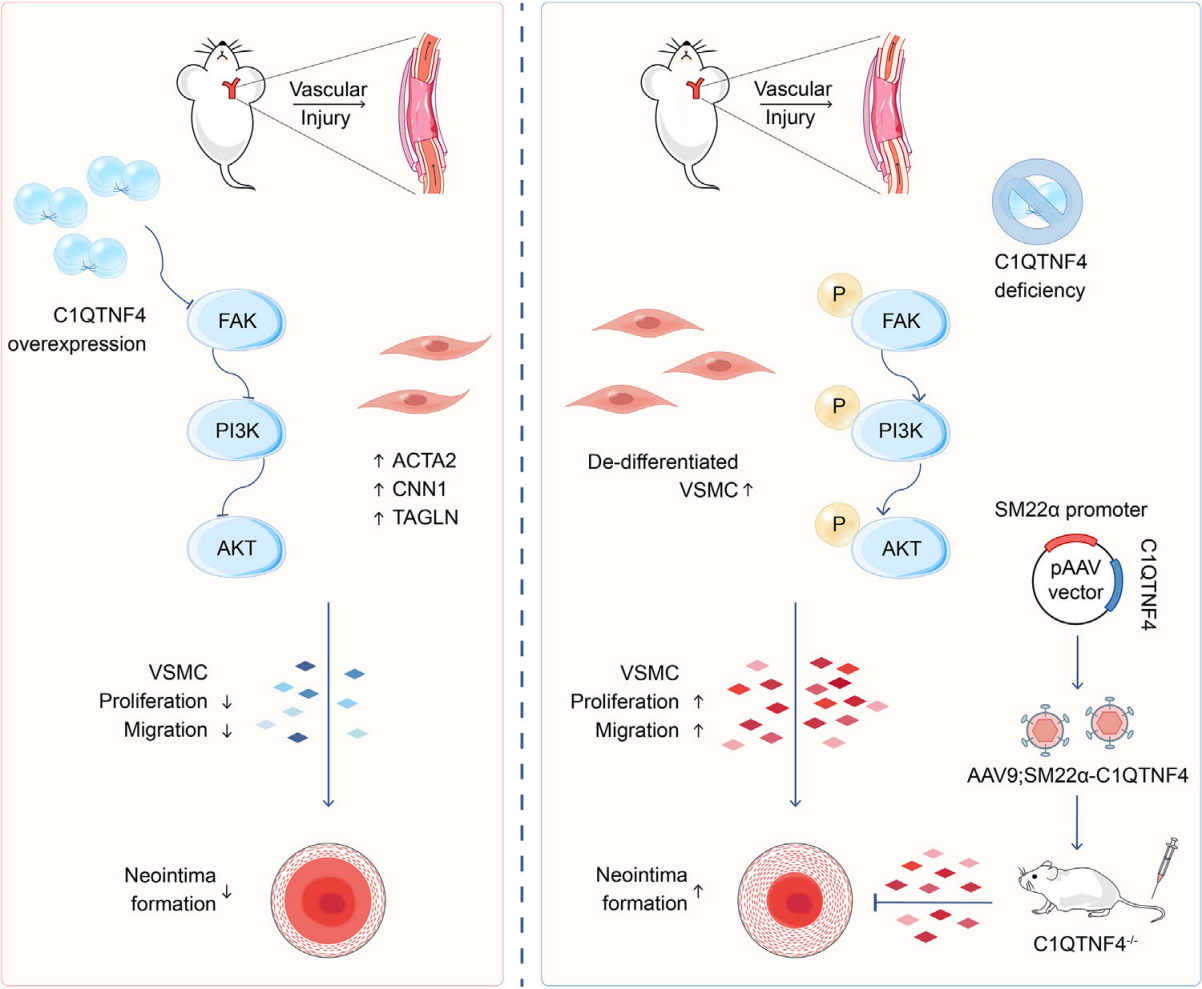
The proposed model by which C1QTNF4 impedes the FAK/PI3K/AKT pathway in the progression of vascular remodelling after vessel injury.

Our results indicated that C1QTNF4 negatively regulates FAK/PI3K/AKT in vitro. Nevertheless, to determine whether C1QTNF4 generally mediates VSMC proliferation and migration through FAK/PI3K/AKT, we assessed this pathway in vivo. On the 14th day after the establishment of the rat balloon injury model, C1QTNF4 protein expression levels, as well as the activation level of the FAK/PI3K/AKT pathway, were detected by immunoblotting (Figure 6A). The western blot results revealed that the transfected adenovirus was expressed in the injured vessels, and C1QTNF4 indeed markedly suppressed the FAK/PI3K/AKT pathway. The overexpression of C1QTNF4 greatly reduced the phosphorylation of FAK, reduced the ratio of p‐PI3K to PI3K and eventually inhibited PI3K activation (Figure 6B). We then used the FAK inhibitor defactinib in a C1QTNF4‐knockout mouse wire‐injury model. The western blot analysis results showed that the activation of the FAK/PI3K/AKT signalling pathway caused by C1QTNF4 deficiency could be suppressed by defactinib (Figure 6C). Collectively, these results reveal that C1QTNF4 inhibits VSMC proliferation and migration by negatively regulating the FAK/PI3K/AKT pathway to protect the intima from abnormality formation (Figure 7).

## 4 DISCUSSION

Vascular remodelling is an adaptive process of blood vessels to long‐term haemodynamic changes and an essential pathophysiological state in many vascular and circulatory diseases. The extensive plasticity of VSMCs makes them susceptible to various stimuli, resulting in hyperplasia and migration.^26^ To date, studies have explored the mechanism by which abnormal cellular behaviour leads to neointimal formation and eventually results in major adverse cardiovascular events (MACEs), vascular restenosis after PTCA, etc.^27^, ^28^, ^29^ However, the underlying molecular mechanism by which the proliferation and migration of VSMCs are regulated is not fully understood. This work identifies a novel mechanism for this remodelling process. Our in vitro and in vivo experiments demonstrate that C1QTNF4, an adipocytokine with a unique structure, is vital for circumventing abnormal VSMC proliferation and migration to maintain vascular morphology. In contrast, C1QTNF4 knockout greatly aggravates cellular hyperplasia/invasion and contributes to neointimal formation in injured arteries. Furthermore, we identified that C1QTNF4 ameliorates abnormal VSMC proliferation and migration by suppressing FAK/PI3K/AKT signalling, which can attenuate angiostenosis in vivo.

The C1QTNF family is characterised by its highly conserved C‐terminal complement C1q globular domain.^30^ Before our investigation of C1QTNF4, previous studies found that the physiological functions of C1QTNFs are highly diversified. The metabolic processes these adipocytokines are involved in range from fatty acid oxidation to glucose metabolism. A considerable body of literature shows that the C1QTNF family is closely associated with cardiovascular disease. Among the C1QTNFs, C1QTNF4 is the only member with two C1q globular domains and has previously been reported to function in the hypothalamus and in tumour cell survival.^16^, ^17^ Our previous studies reported that the globular domain determines the function of C1QTNFs.^15^, ^16^ So, it is reasonable to make a conjecture that the two C1q globular domains in C1QTNF4 are functionally enhanced. To date, there is very little published literature on C1QTNF4 in cardiovascular disease. Considering the unique structure of C1QTNF4 and the relationship between cardiovascular disease and some of the C1QTNF family members, we investigated the role of C1QTNF4 in blood vessels. Intriguingly, unlike the gain‐of‐function effect in tumour cells, we observed an inhibitory effect against VSMC processes in several kinds of experiments. First, we detected a decrease in serum C1QTNF4 levels in patients with carotid stenosis. However, one of the limitations of the human data is that the sample size is relatively small. We next detected C1QTNF4 and α‐SMA expression in human normal renal artery and renal artery plaque. The results suggest that C1QTNF4 is mainly expressed in VSMC and rarely expressed in the area of plaque. Then, we explored the influence of C1QTNF4 on VSMC migration and proliferation in vitro. The results reveal that C1QTNF4 dose‐dependently inhibits VSMC migration and proliferation. Further, we continued to examine the phenotypic switching of VSMCs. Our results indicated that C1QTNF4 attenuates contractile VSMCs dedifferentiating into synthetic VSMCs. And drivers of VSMC phenotype modulation, such as PDGF, TGFβ or FGF, do not alter C1QTNF4 expression. In addition, we also studied the potential impact of C1QTNF4 on apoptosis in vitro. Our results indicate that C1QTNF4 was not involved in apoptosis. Thus, all monitoring methods pointed to an inhibitory effect of C1QTNF4 on cell proliferation, and the effect was time‐ and dose‐dependent.

Our in vitro study confirmed that recombinant C1QTNF4 protein suppresses the proliferation and migration of VSMCs. However, we wanted to confirm the effect of C1QTNF4 on vascular remodelling. In vitro experiments alone are not convincing enough to support our hypothesis. We investigated whether C1QTNF4 is abnormally activated in vascular lesions in a variety of animal models. In the rat balloon injury model, we found that Ad‐C1QTNF4‐infected rat carotid arteries had less neointima formation than Ad‐LacZ‐infected rat arteries. While the tunica media and tunica adventitia remained almost unchanged. Notably, the recombinant adenovirus used in the study contained human C1QTNF4. Previous studies have identified that C1QTNF4 sequences in different vertebrates are highly evolutionarily conserved.^16^ Human and mouse C1QTNF4 share over 90% homology in amino acid sequence, which implies similar roles in different species. However, the adenoviral vectors used in our balloon injury model do not integrate into the host genome, making it difficult to fully simulate physiological conditions. To help address this issue, we used a previously published C1QTNF4‐TG mouse model to mimic the process of VSMC‐mediated repair and remodelling.^20^ Our preliminary results showed that the phenotype of the transgenic mice used for C1QTNF4 guide wire injury was clearly identified, and the introduced C1QTNF4 was found to be expressed in the common carotid artery of the mice. The hyperplasia of the tunica intima in C1QTNF4‐TG mice caused by guide wire injury was markedly attenuated compared with that in WT mice. Also, the neointima/media ratio positively correlated with the neointimal area. Together, we concluded that C1QTNF4 could indeed reduce vascular remodelling to some extent. In addition, we detected changes in C1QTNF4 levels over time after wire‐injury in WT and C1QTNF4‐TG mice. Intriguingly, the results show that C1QTNF4 levels accumulate during the early phase after injury and decrease over time. Whereas, C1QTNF4 levels in patients with carotid stenosis were lower than those in healthy subjects, as presented in Table 1. A possible explanation for this phenomenon is that C1QTNF4 could rapidly be mobilised and recruited to the injured site at the endothelial surface in response to acute changes. Next, we generated a novel C1QTNF4‐knockout mouse model. The intimal thickening of C1QTNF4‐deficient vessels was significantly more severe than that of control vessels. Furthermore, we generated a novel AAV vector to assess the specific role of C1QTNF4. We used SM22α to ensure specific expression in smooth muscle cells, so that the expression of C1QTNF4 in vascular smooth muscle cell was restored in C1QTNF4‐knockout mice. Neointimal formation can only be partially alleviated when restored the expression of C1QTNF4 in VSMCs of the C1QTNF4‐knockout mice. The current results implied that VSMCs may affected by serum and local C1QTNF4 levels when remodelling the vessel wall. These results suggest that C1QTNF4 affects VSMCs through both blood circulation and intracellular generation and may provide new insights for the development of vessel stenosis disease treatments. However, future studies are needed to further explore the secretion and transformation patterns of C1QTNF4. Besides, in order to further verify the role of C1QTNF4 in vascular wall, we explored whether other cellular constituents were involved in the process. Our immunofluorescence staining results illustrated that C1QTNF4 was mainly expressed in VSMC and no significant changes were seen in endothelial, T cell or macrophage. In addition, we also analysed metabolism in C1QTNF4‐TG and KO mice. No significant changes were found in weight gain or lipid profile.

The essential issue of this study is the molecular mechanism by which C1QTNF4 regulates the proliferation and migration of VSMCs. After discovering that C1QTNF4 suppresses vascular remodelling in genetically modified mice, we continued to explore the mechanisms underlying the effects of C1QTNF4 on tunica intima thickening. C1QTNF family members have been systematically studied as inflammatory factors and fat factors.^10^, ^15^, ^31^ Notably, C1QTNF1, C1QTNF3 and C1QTNF9 have been reported to be involved in the signal transduction of cell migration or proliferation.^14^, ^32^, ^33^ As outlined above, C1QTNF4 attenuates abnormal vascular injury repair. Nevertheless, it is unknown how C1QTNF4 regulates the proliferation and migration of VSMCs through interactions with other signalling molecules. We performed RNA sequencing to find the potential underlying mechanism. There were alterations in the expression of PI3K/AKT and MAPK signalling pathways proteins. The PI3K/AKT and MAPK signalling pathways are classical pathways for smooth muscle cell proliferation and migration during carotid artery injury. We found that C1QTNF4 acts through the PI3K/AKT pathway, while the MAPK pathway remains unchanged. We next explored the upstream of PI3K. FAK is an intracellular signalling protein that plays an important role in cell proliferation and migration.^34^ FAK can be phosphorylated at multiple tyrosine residues, in which Tyr397 phosphorylation produces an SH2 binding site that facilitates binding to PI3K P85.^35^ We observed that changing the level of C1QTNF4 in VSMCs affects the phosphorylation of FAK, suggesting that C1QTNF4 is a FAK inhibitor and alleviates abnormal VSMC behaviour. This may be due to the involvement of C1QTNF4 in the assembly and release of focal adhesions. Our in vitro and in vivo experiments showed that the overexpression of C1QTNF4 in VSMCs inhibits FAK and PI3K phosphorylation and thereby inhibits downstream AKT phosphorylation.

However, one of the limitations of the current study is that we have not yet found the receptor for C1QTNF4 in VSMC. Efforts to search for its candidate receptors are underway. Currently, FAK inhibitors are mostly used in the treatment of cancer,^36^, ^37^, ^38^, ^39^ and a few are used for the treatment of other diseases, such as liver and lung fibrosis.^40^, ^41^ In this study, we observed the unique role that C1QTNF4‐mediated downregulation of the FAK/PI3K/AKT pathway plays in reducing the abnormal proliferation and migration of VSMCs. The vasoprotective action of C1QTNF4 offering important translational perspectives. AAV‐based targeted therapy of C1QTNF4 may provide ideas for the treatment of in‐stent restenosis and stenosis after arterial injury caused by interventional procedures. In rats and mice, we observed that C1QTNF4 could significantly ameliorate neointima formation. AAV‐based targeted therapy of SMC markedly reduced vascular remodelling after wire injury in C1QTNF4‐KO mice. These provide a relevant proof of principle for the therapeutic approach. On the basis of our study, we envisage possibility of using C1QTNF4‐based therapy during transarterial procedures, to protect the vessel from severe stenosis due to intimal hyperplasia after arterial injury. Further extensive experimentations will be needed to assess the C1QTNF4 associate vasoprotective drug's pharmacological profiles, route of administration, and the safe dose.

In summary, the results of the current study reveal that C1QTNF4 inhibits the proliferation and migration of VSMCs caused by vascular injury and protects blood vessels from intimal hyperplasia to some extent. Changing the level of C1QTNF4 affects the progression of vascular remodelling. We have demonstrated that C1QTNF4 suppresses the proliferation and migration of VSMCs, that is, neointima formation, by downregulating the FAK/PI3K/AKT signalling pathway. Our results reveal that C1QTNF4 may be a new drug target and shed light on novel gene therapies for vascular stenosis diseases.

## CONFLICT OF INTEREST STATEMENT

The authors declare no conflicts of interest.

## Supporting information

Supporting information
