# Supplementary material for: C1q/TNF‐related protein 4 mediates proliferation and migration of vascular smooth muscle cells during vascular remodelling
Source: Clin Transl Med. 2023 May 23;13(5):e1261. doi: 10.1002/ctm2.1261 (PMC10206021; doi:10.1002/ctm2.1261)
Supplement: Supplementary file 1 — Supporting information [file CTM2-13-e1261-s001.docx]

Supplementary Information.

**C1q/TNF-Related Protein 4 Mediates Proliferation and Migration of Vascular Smooth Muscle Cells During Vascular Remodeling**

Jingying Liu^1,2^, Xingbo Long^1,3^, Hexin Li^4^, Qiuxia Yan^1,3^, Lu Wang^5^, Ziyu Qin^1^, Hong Zhang^1^*

^1^State Key Laboratory of Vascular Homeostasis and Remodeling, The lnstitute of Cardiovascular Sciences, School of Basic Medical Sciences, Peking University Health Science Center, Beijing, China; Beijing 100191, China.

^2^Department of Cardiology, Fuwai Hospital, Chinese Academy of Medical Sciences & Peking Union Medical College/National Center for Cardiovascular Diseases; Beijing 100037, China.

^3^National Center of Gerontology, Beijing Hospital; Beijing 100730, P.R China.

^4^Biological Sample Management Center, Beijing Hospital; Beijing 100730, P.R China.

^5^Center for Human Disease Genomics, Peking University Health Science Center; Beijing 100191, China.

*Corresponding authors: Hong Zhang (zhanghong@bjmu.edu.cn)

**Contents for Supporting Information**

**Supplementary Figures**


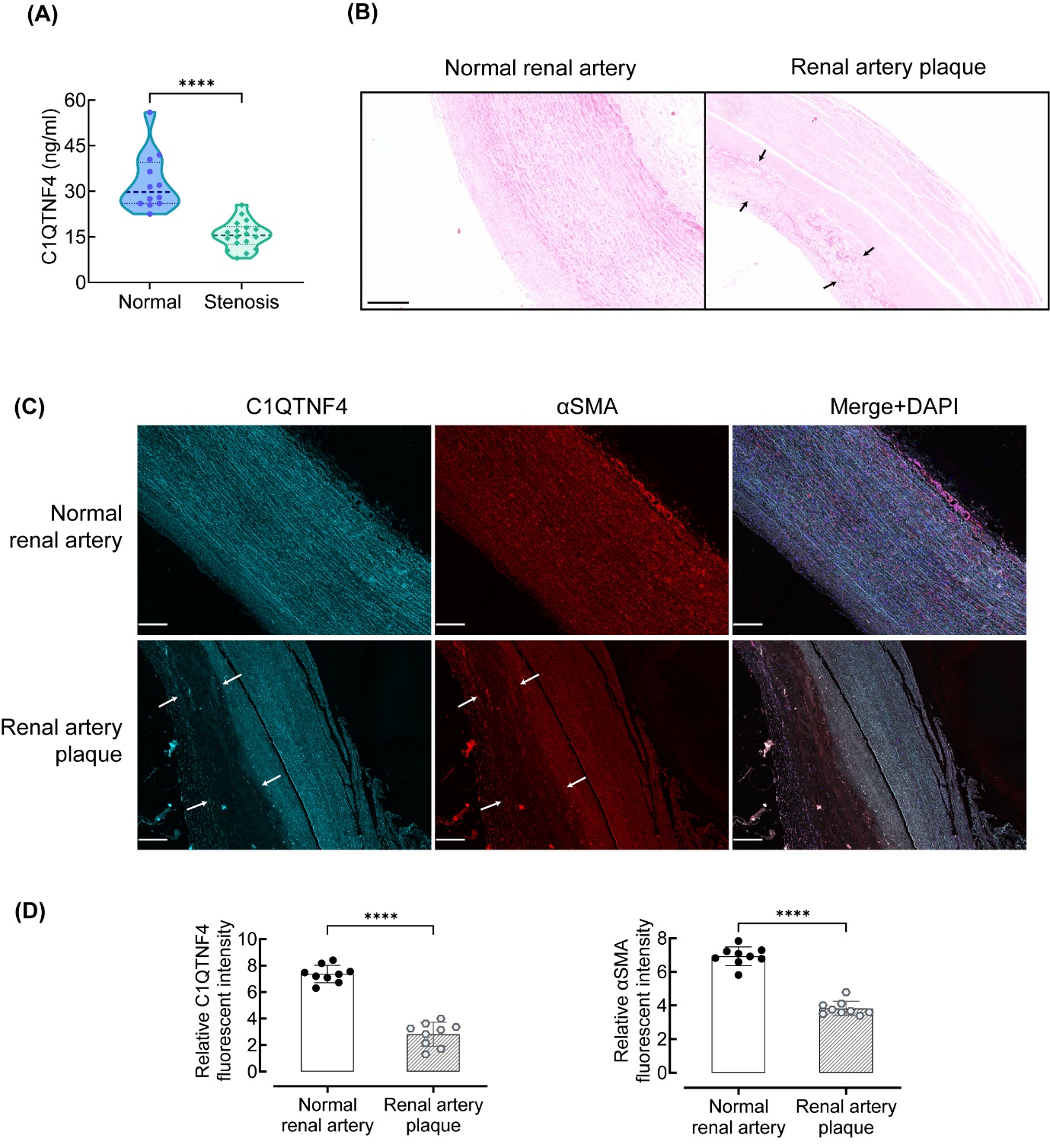


**Supplementary Figure 1. C1QTNF4 levels in patients with or without arterial stenosis.**

(A) Plasma C1QTNF4 level in patients with normal carotid artery (n=12) and patients with carotid stenosis (n=18). Plasma concentration of C1QTNF4 was measured by ELISA system. (B) Representative HE staining of human normal renal artery and renal artery plaque. Scale bar, 300 μm. Arrow indicates the area of plaque. (C) Immunofluorescent staining of human normal renal artery and renal artery plaque. Slides were simultaneously stained with a multiplex quantitative immunofluorescence (QIF) panel containing C1QTNF4 (cyan), α-SMA (red), and DAPI (blue). Scale bar, 300 μm. Arrow indicates the area of plaque. (D) Quantification of the C1QTNF4 and α-SMA expression in vascular smooth muscle cells in the injured arteries (±SEM, n=9). ****p<.0001.


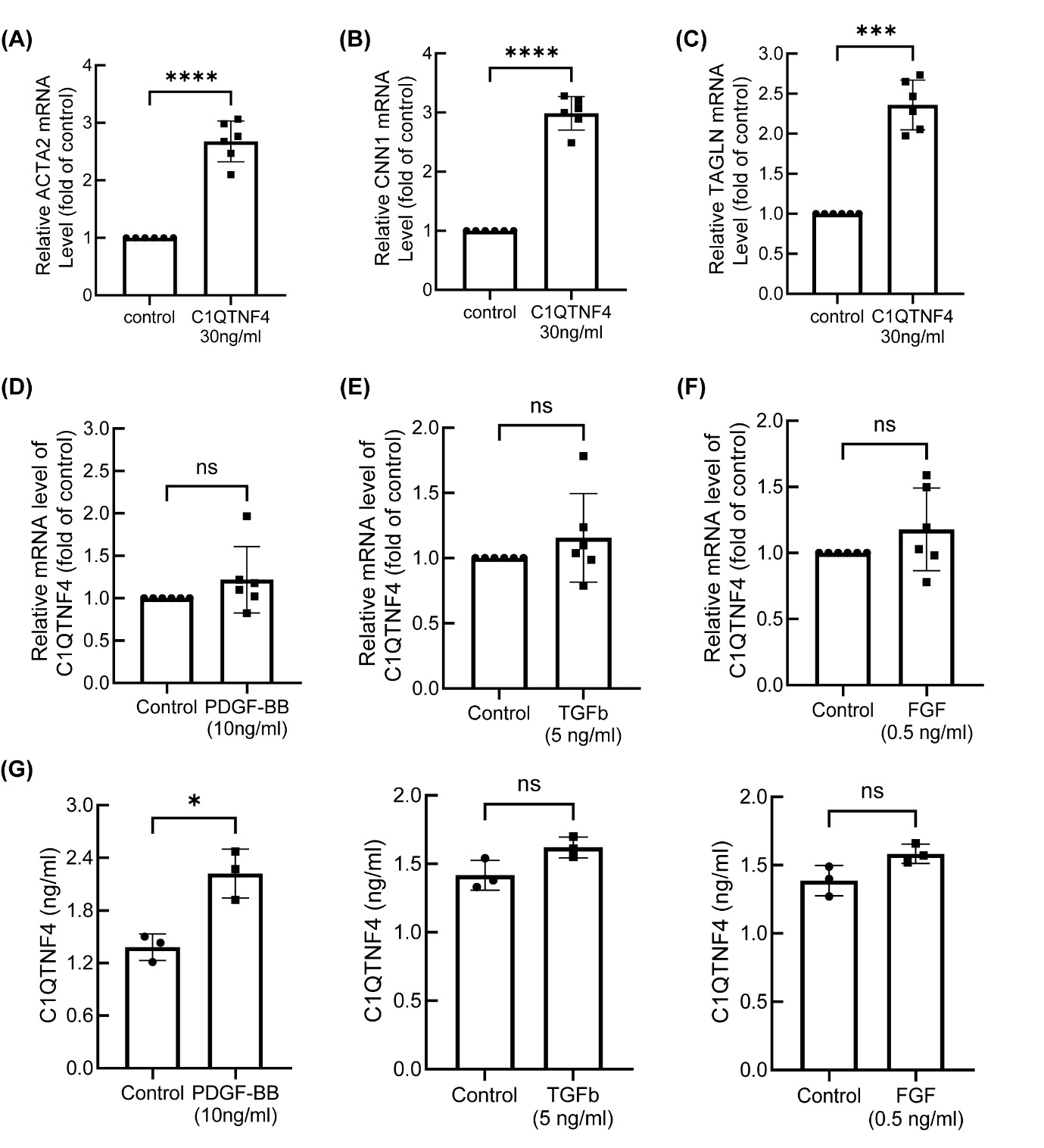


**Supplementary Figure 2. C1QTNF4 expression regulates VSMCs phenotype.**

(A-C) VSMCs were treated with or without 30 ng/ml recombinant C1QTNF4 protein for 24 hours. Relative mRNA level of the ACTA2(α-SMA), CNN1(calponin), and TAGL(SM22a) in VSMCs were measured. C1QTNF4 increases VSMC contractile marker gene expression (±SEM, n=6). (D) Relative mRNA level of C1QTNF4 in VSMCs with or without PDGF-BB (10ng/ml) stimulation for 12 hours. (E) Relative mRNA level of C1QTNF4 in VSMCs with or without TGFβ (5ng/ml) stimulation for 24 hours. (F) Relative mRNA level of C1QTNF4 in VSMCs with or without FGF (0.5ng/ml) stimulation for 24 hours. ±SEM, n=6, two-tailed Student’s t-test. (G) The expression of C1QTNF4 in culture supernatant of VSMCs with or without PDGF-BB, TGFβ or FGF stimulation were measured by ELISA system (±SEM, n=3). *p<.05. ***p<.001. ****p<.0001. NS, no statistical significance.


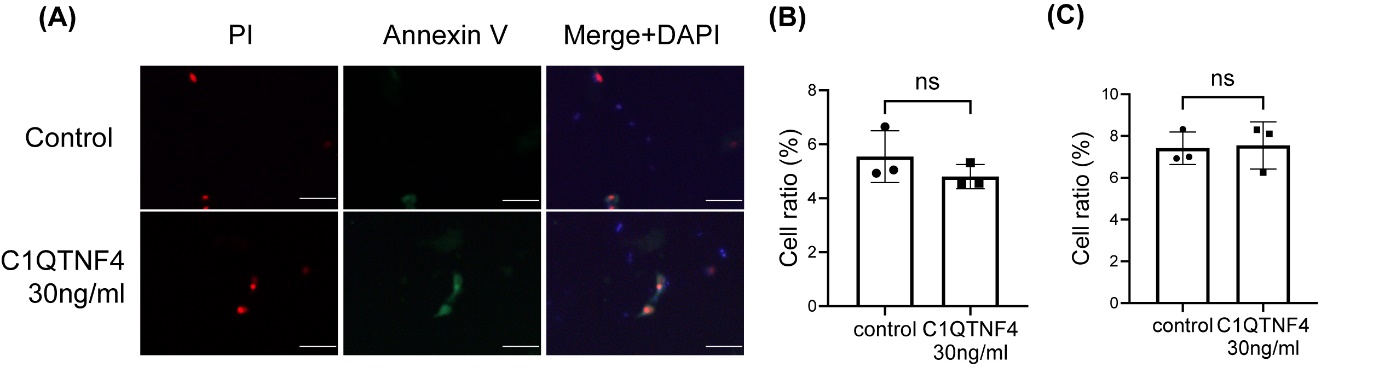


**Supplementary Figure 3. C1QTNF4 does not affect the apoptosis or necrosis of VSMCs.**

(A) Immunocytochemistry for propidium iodide (PI, red), annexin V (green), or a merged channel with DAPI (blue for nuclei) in VSMCs treated with or without 30 ng/ml recombinant C1QTNF4 protein for 24 hours. Scale bar, 100 μm. (B-C) Flow cytometry results show apoptotic cell (B) and necrotic cell (C) ratio of VSMCs treated with or without 30 ng/ml recombinant C1QTNF4 protein for 24 hours (±SEM, n=3). NS, no statistical significance.


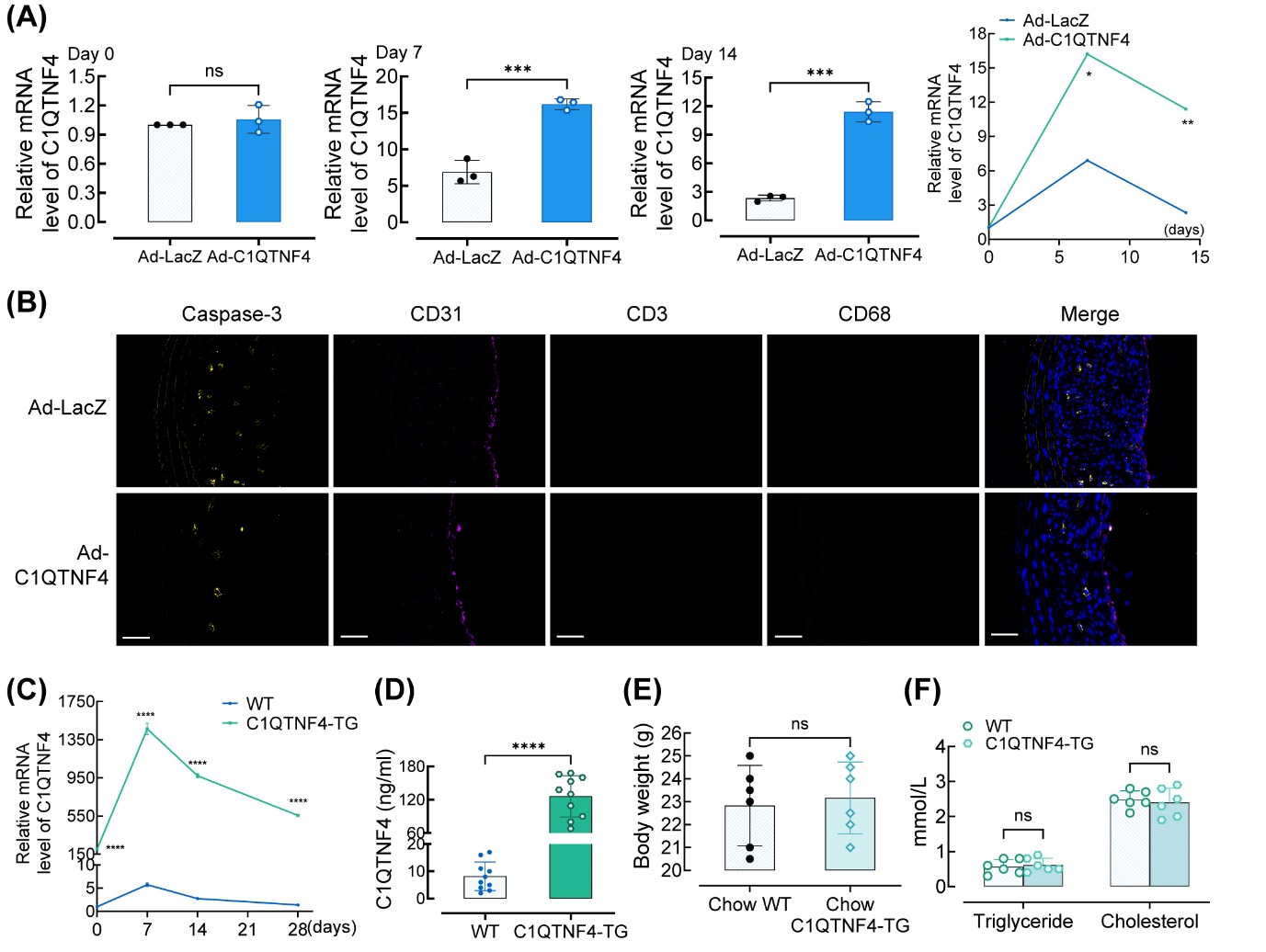


**Supplementary Figure 4. Overexpression of C1QTNF4 in rat and mouse models, and potential confounding factors.**

(A) Polymerase chain reaction (PCR) of rats common carotid arteries infected with Ad-C1QTNF4 or Ad-LacZ at 0, 7 and 14 days after balloon injury. The content of C1QTNF4 in the control group on 0 day was set as 1, and the rest of the groups were analyzed by fold changes (±SEM, n=3). (B) Representative immunofluorescent staining of Ad-C1QTNF4-infected and Ad-LacZ-infected rat common carotid arteries at 14 days after balloon injury. Slides were simultaneously stained with a multiplex quantitative immunofluorescence (QIF) panel containing caspase-3 (yellow), CD31 (violet), CD3 (red), CD68 (green) and DAPI (blue). Scale bar, 30 μm. (C) PCR of C1QTNF4-TG and WT mice common carotid arteries on the 0, 7^th^, 14^th^ and 28^th^ days after wire injury. The content of C1QTNF4 in the control group on 0 day was set as 1, and the rest of the groups were analyzed by fold changes. (D) The expression of C1QTNF4 in the blood circulation of WT and C1QTNF4-TG mice measured by ELISA system (±SEM, n=10). (E) Body weights of WT and C1QTNF4-TG mice on chow diet at 14 days after carotid ligation (±SEM, n=6). (F) Quantitative measurements of serum total cholesterol and triglyceride levels in WT and C1QTNF4-TG mice on the chow diets at 14 days after carotid ligation (±SEM, n=6). *P<0.05, **P<0.01, ***P<0.001, ****P<0.0001; NS indicates no significance.


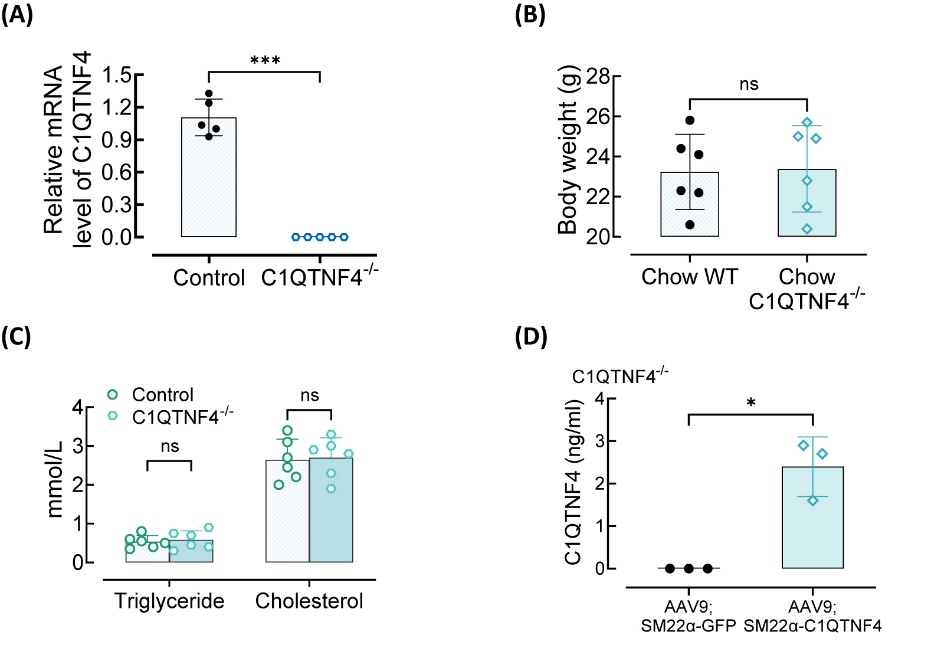


**Supplementary Figure 5. The C1QTNF4-knockout mouse model and potential confounding factors.**

(A) Relative mRNA level of C1QTNF4 in the common carotid arteries of control mice and C1QTNF4-knockout (C1QTNF4^-/-^) mice (±SEM, n=5). (B) Body weights of WT and C1QTNF4-knockout mice on chow diet at 14 days after carotid ligation (±SEM, n=6). (C) Quantitative measurements of serum total cholesterol and triglyceride levels in WT and C1QTNF4-knockout mice on the chow diets at 14 days after carotid ligation (±SEM, n=6). (D) The expression of C1QTNF4 in the blood circulation of C1QTNF4^-/-^; AAV9; SM22α-GFP mice and C1QTNF4^-/-^; AAV9; SM22α-C1QTNF4 mice were measured by ELISA system (±SEM, n=3). *p<.05, ***p<.001. NS, no statistical significance.


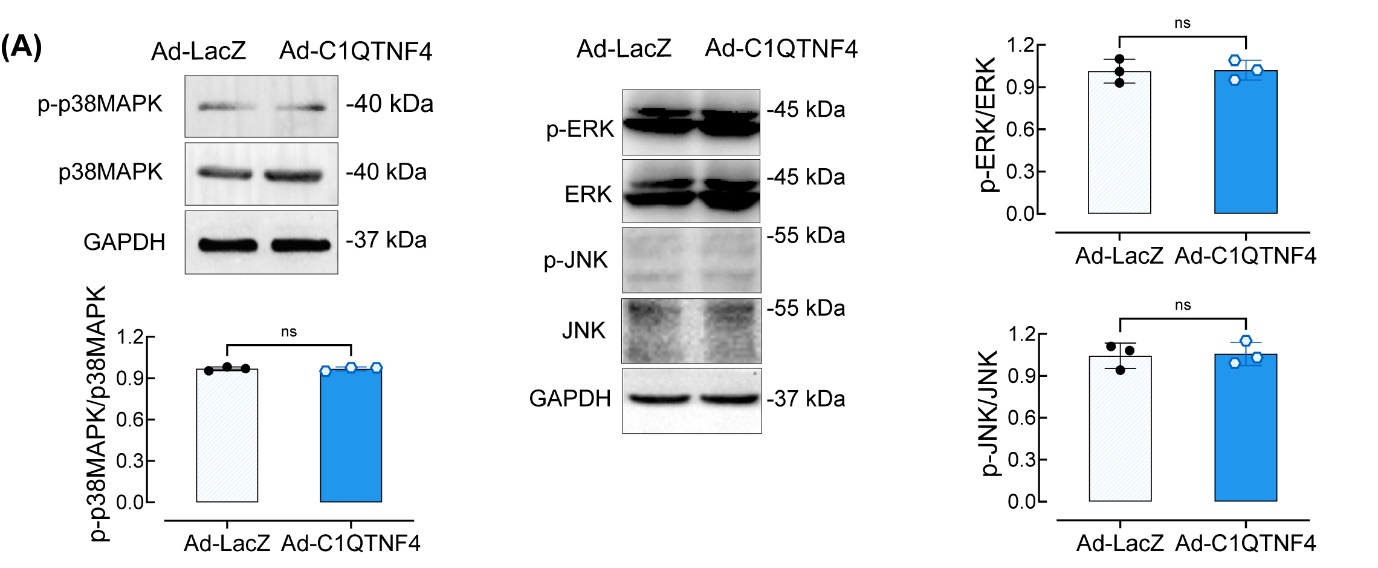


**Su****pplementary Figure 6.** **Potential mechanisms by which C1QTNF4 regulates VSMC proliferation and migration.**

(A) Western blot of VSMCs infected with Ad-C1QTNF4 or Ad-LacZ for p-p38MAPK, p38MAPK, p-ERK, ERK, p-JNK, JNK and GAPDH as the loading control. p-p38MAPK/p38MAPK, p-ERK/ERK, p-JNK/JNK was assessed (±SEM, n=3). NS, no statistical significance.


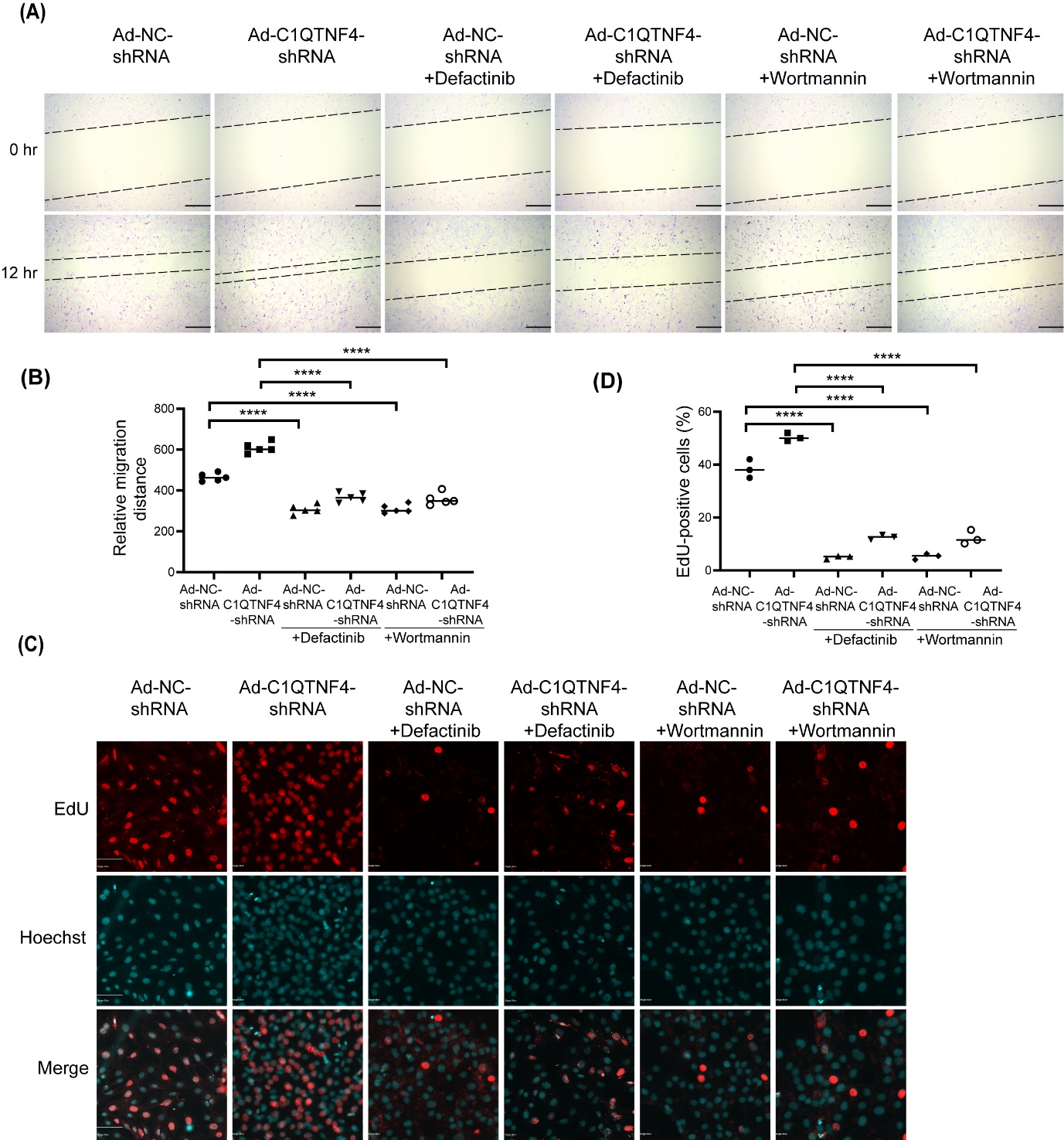


**Supplementary Figures 7.** **FAK and PI3K inhibitors in deficient C1QTNF4 VSMCs.**

(A-B) Representative images of the migration assays treated with or without FAK or PI3K inhibitor at 0 hour and after 12 hours culture. The dotted line indicates the wound edge. Scale bar, 50 μm. The mean migration distance of VSMCs was quantified at 12 hours after scratching (±SEM, n=5). (C-D) Representative images of EdU incorporation in VSMCs incubated with or without FAK or PI3K inhibitor for 36 h. Nuclei were stained with Hoechst 33342 (blue) and the proliferating cells were labeled with EdU (red). Scale bars, 50 μm. Percentages of EdU-positive cells were calculated (±SEM, n=3). ****P<0.0001

# Supplementary Table

**Supplementary Table. 1 |** Demographic details of the human renal arteries samples.

| Subjects | Diagnosis | Age | Gender | Ethnicity | Other Information |
| --- | --- | --- | --- | --- | --- |
| 1 | Brain Death | 38 | Male | Asian | normal renal arteries |
| 2 | Brain Death | 50 | Female | Asian | normal renal arteries |
| 3 | Brain Death | 40 | Male | Asian | normal renal arteries |
| 4 | Brain Death | 41 | Male | Asian | renal artery plaque |
| 5 | Brain Death | 62 | Male | Asian | renal artery plaque |
| 6 | Brain Death | 50 | Female | Asian | renal artery plaque |

# Supplementary Method

***Human sample collection.***

Serum specimens were collected between 2019 and 2020. Healthy Asian subjects or patients with carotid stenosis >70% were included in the study. Participants were excluded from the study if any of the following conditions were present: 1) underwent carotid endarterectomy or carotid artery stenting, 2) connective tissue diseases or immunological diseases, 3) mental disorders, 4) asthma or COPD, 5) hematological disorders, 6) thyroid diseases, 7) malignancies, or 8) abnormal liver function. All procedures followed were in accordance with Peking University guidelines. Written informed consent from all individual participants was obtained. Normal renal arteries and renal artery plaque were obtained from six donors after brain death between 2019 and 2020. The baseline characteristics of the donors are listed in Supplementary Table 1. Consent for renal donation was obtained from the patients prior to death. Also, full written consent was given by the family to use the donor organ for transplantation and research purposes. Human studies received approval by the Institutional Review Board of Beijing Hospital (No.2017BJYYEC-108-05) and the procedures followed were in accordance with Peking University Health Science Center and Beijing Hospital guideline. The study conformed to the Declaration of Helsinki. Experiments were conducted in compliance with all relevant ethical regulations.

***Recombinant Adenovirus Construction and RNA Interference.***

The coding sequence of human full-length C1QTNF4 protein (HA or Myc tagged at the 5’-end) was subcloned into the adenovirus shuttle vector pAdlox. It was cotransfected with replication-defective adenovirus genomic DNA into engineered CRE8 cells, which stably express the Cre recombinase enzyme for homologous recombination. The BD Knockout RNAi system from BD Clontech was used to generate the recombinant siRNA adenovirus. The hairpin shRNA sequence was encoded by the U6 promoter and was recombined into the adenoviral genome after the effectiveness of gene silencing was verified. An adenovirus carrying green fluorescent protein (Ad-GFP) was used as a negative control. A nontargeting shRNA adenovirus was used as a control for C1QTNF4 shRNA adenovirus (Ad-NC-shRNA).

***Cell Culture***

Approximately 200 g of the thoracic aorta of Sprague-Dawley rats was taken. The tunica media was cut into small pieces of 1.5~2.0 mm^3^ and spread evenly in a culture flask. DMEM containing 20% FBS was added. After incubation for 4 h, the culture flask was inverted to submerge the tissue and statically cultured for 10~14 d. When the SMCs reached 90% confluence, they were passaged with 0.125% trypsin. The purity of the VSMCs was identified by histochemical staining with anti-α-SMA. SMC cultures with purities greater than 95% within 9 generations were used in the experiments.

***Preparation of the Recombinant Human C1QTNF4 (rhC1QTNF4) Protein.***

The recombinant C1QTNF4 protein was expressed and purified in our laboratory. Briefly, to generate the prokaryotic recombinant protein, the pET32a-C1QTNF4 plasmids were transfected into DE3 Escherichia coli. The expression of C1QTNF4 was induced by isopropyl-β-d-thiogalactoside. After 10 h, the cells were collected and resuspended in lysis buffer (20 mM PIPES, 500 mM NaCl and 20 mM imidazole). The lysate was centrifuged, and the supernatant was purified by nickel affinity chromatography. The purified protein was further purified by diethyl-aminoethanol ion-exchange chromatography. The level of LPS in the purified protein samples was determined by tachypleus amebocyte lysate produced in Xiamen, China.

***Quantification of VSMC Proliferation.***

EdU staining, MTT assay and cell counting were used to evaluate the proliferation of VSMCs and performed by conventional methods. In EdU (5-ethynyl-2′-deoxyuridine) incorporation assay, VSMCs in the logarithmic growth phase were seeded in 96-well plates. After transfection, cells were incubated with EdU in medium (50 μM) for 2 h. Then, 4% paraformaldehyde was used to fix cells for 30 min. After washing in 0.5% Triton X-100, the VSMCs were stained with Hoechst 33342 (5 mg/ml) for 30 min at room temperature. EdU staining was performed using a Click-iT Plus EdU Alexa Fluor 488 Imaging Kit (Thermo Fisher).

***VSMC Migration Assays.***

VSMC migration was assessed by scratch assay, transwell assay and confocal microscopy, using conventional methods. The confocal microscopy involved the fixation of the VSMCs with 4% paraformaldehyde and extraction of membrane proteins with 0.5% Triton-X10. The stress fiber of cells was shown by rhodamine-phalloidin staining and observed by confocal microscopy (Zeiss LSM 510).

***Rat and Mouse Carotid Artery Injury Models.***

Sprague-Dawley rats weighing 350 to 450 g, C1QTNF4-/- mice, sex-matched littermate control mice, C1QTNF4-TG mice, and sex-matched littermate WT mice weighing 19 to 25 g were used in these experiments. All the animals were experimented at eight weeks old. The sex of the rats or mice used in the experiment was half male and half female. Rats were anesthetized with 100% O_2_/4% isoflurane, and the whole process was maintained with 100% O_2_/2% isoflurane. Balloon (Medtronic, Minneapolis, MN) induced carotid artery injury was performed. The common carotid artery lumen was injected with adenovirus (2×10^8^ pfu). After 15 minutes of incubation, the virus solution was removed, and the proximal end of the external carotid artery was ligated. Likewise, mice were anesthetized, and wire-induced carotid artery injury was performed. Briefly, a metal guide wire with a diameter of 0.38 mm (No. C-SF-15-15, Cook, Bloomington, Indiana) was inserted by approximately 5 mm, rotated 5 times to rub against the vascular wall, left in place for 1 min to denude and dilate the artery and then removed. After the operation, tramadol (10 mg/kg) was injected into the caudal vein of the animal for analgesia. At the end of the experiments, euthanasia was performed using carbon dioxide followed by cervical dislocation. All animal procedures followed protocols approved by the Animal Care and Use Committee at Peking University [approval number LA2014110], and conformed to the guidelines from Directive 2010/63/EU of the European Parliament on the protection of animals used for scientific purposes and the NIH Guide for the Care and Use of Laboratory Animals.

***AAV9; SM22α-GFP and AAV9; SM22α-C1QTNF4 mouse model establishment.***

AAV9; SM22α-GFP and AAV9; SM22α-C1QTNF4 vectors were generated by Packgene (Guangzhou, China). AAV9; SM22α-GFP and AAV9; SM22α-C1QTNF4 were specially expressed in SMC by SM22α. Six-week-old C1QTNF4^-/-^ mice were randomly selected for AAV9; SM22α-GFP injections (1×10^13 GC each mouse, diluted into 50-100 μl, tail vein injection) or AAV9; SM22α-C1QTNF4 (1×10^13 GC each mouse, diluted into 50-100 μl). Two weeks after injection, carotid artery PCR was performed to observe the C1QTNF4 expression. Also, two weeks post AAV9 injection, carotid artery wire injury surgery was performed. Then after 2 weeks, the animals were sacrificed, and the carotid arteries were harvested for paraffin embedding, sectioning, and HE staining. Procedures followed protocols approved by the Animal Care and Use Committee at Peking University [LA2014110] and the NIH Guide for the Care and Use of Laboratory Animals.

***Hematoxylin and Eosin Staining.***

At 0-4 weeks postoperatively, some mice and rats were fixed with 4% formaldehyde pressure perfusion through the arterial system and embedded in sections. HE staining were performed to evaluate neointimal hyperplasia.

***Multiplex immunofluorescence and immunofluorescent detection.***

Cross-sections of formalin-fixed, paraffin-embedded tissue (The rat carotid artery, mouse carotid artery, human normal renal artery, and human renal artery plaque) were used for a multiplex immunofluorescence. The samples were incubated with antibody against Ki67, C1QTNF4, CD3, caspase-3, CD31, alpha smooth muscle actin, CD68, or elastin. For antibody detection, the Opal 5-Color Manual IHC Kit (PerkinElmer) was used. Each antibody was assigned to one of the fluorophores Opal 520, Opal 570, Opal 620 and Opal 690. All slides were cover slipped using Vectashield Hardset mounting media with DAPI (Vector Laboratories). Multiplex immunofluorescence scans were captured by a PerkinElmer Vectra Polaris. Images were analyzed using inForm Cell Analysis software (Perkin Elmer).

***RNA sequencing****.*

Carotid arteries from WT and C1QTNF4-TG mice were collected at 14 days after wire injury. After removed the tunica adventitia, the tissues were used for RNA sequencing which was performed by novelbio (Shanghai, China). In brief, total RNAs (n=5 for each group) were extracted with TRIzol Reagent (Invitrogen). The cDNA libraries were constructed for each pooled RNA sample using the NEBNext® Ultra™ Directional RNA Library Prep Kit for Illumina according to the manufacturer’s instructions. Libraries were paired-end sequenced (2 × 150 bp reads) on Illumina HiseqXTen platform. During the experiment, investigators were blinded to samples information. Before read mapping, clean reads were obtained from the raw reads by removing the adaptor sequences, reads with >5% ambiguous bases (noted as N) and low-quality reads containing more than 20 percent of bases with qualities of <20. The clean reads were then aligned to mouse genome (NCBI GRCm38.p4; https://www.ncbi.nlm.nih.gov/genome/52?genome_assembly_id=992563) using the hisat2. Samples were subjected to quality control by examining the percentage of reads uniquely mapping to the genome, the percentage of reads mapping to known protein coding sequences, and the number of genes with 90% base coverage. Differentially expressed genes (DEGs) were identified by counting the number of reads mapping to each gene from NCBI GRCm38.p4 using Htseq-count. Reads Per Kilobase per Million mapped reads (RPKM) were analyzed using Stringtie. To remove batch effect, we employed the “removeBatchEffect” function from R package “limma”.

*Pathway enrichment analysis*

Gene Set Variation Analysis (GSVA) and Gene Set Enrichment Analysis (GSEA) enrichment analysis (http://software.broadinstitute.org/gsea/index.jsp) was used to find out the significant changed pathways between WT and C1QTNF4-TG group according to KEGG database (https://www.genome.jp/kegg/pathway.html) by using the gsea java software (Version 3.0; https://www.gsea-msigdb.org/gsea/datasets.jsp) and R package GSVA respectively. Raw data have been deposited in the National Center for Biotechnology Information Gene Expression Omnibus under no. GSE195630.

***Real-Time Quantitative PCR and Western Blot Analysis.***

Total RNA was extracted using TRIzol reagent (Thermo Fisher Scientific). cDNA was obtained from 1 μg RNA by using the Reverse Transcription Reagent Kit, and real-time PCR was performed with SYBR Green mix (Thermo Fisher Scientific). PCR primers were designed according to the NCBI reference sequences. In western blot analysis, cells or tissues were lysed in cold isotonic lysis buffer containing a protease inhibitor cocktail (Santa Cruz Biotechnology, Santa Cruz, CA, USA). Protein was resolved by electrophoresis on 10-15% SDS-PAGE and transferred onto PVDF membranes, which were incubated with primary antibodies overnight at 4°C with gentle shaking and then with horseradish peroxidase–conjugated secondary antibodies for 1 h. Specific bands were visualized with electrochemiluminescence (ECL) reagents (Thermo Fisher Scientific, Rockford, IL, USA).

# Major Resources Tables

**Animals (in vivo studies)**

| **Species** | **Vendor or Source** | **Background Strain** | **Sex** | **Persistent ID / URL** |
| --- | --- | --- | --- | --- |
| Mouse, mus musculus | Shanghai Model Organisms Center, Inc. | C57BL/6N | Half male and half female | NM-KO-200070  NCBI: 67445 |
| Rat, Rattus Norvgicus | Charles River | Sprague Dawley | Half male and half female | https://www.vitalriver.com/products-services/research-models/2c9f1f26696b40bf01696b647d7b0001 |
| Mouse, mus musculus | Center for Human Disease Genomics, Peking University | C57BL/6N | Half male and half female | Establishment of adipocytokine CTRP4 transgenic mouse. DOI：doi:10.1038/cmi.2016.16 |
| Mouse, mus musculus | Charles River | C57BL/6N | Half male and half female | https://www.vitalriver.com/products-services/research-models/2c9f1f26699fc7810169c2020d210018 |

**Genetically Modified Animals**

| **Species** | **Vendor or Source** | **Background Strain** | **Other Information** | **Persistent ID / URL** |
| --- | --- | --- | --- | --- |
| Mouse, mus musculus | Shanghai Model Organisms Center, Inc. | C57BL/6N | Both M and F used | NM-KO-200070  NCBI: 67445 |
| Mouse, mus musculus | Center for Human Disease Genomics, Peking University | C57BL/6N | Both M and F used | Establishment of adipocytokine C1QTNF4 transgenic mouse. DOI：doi:10.1038/cmi.2016.16 |

**Antibodies**

| **Target antigen** | **Vendor or Source** | **Catalog #** | **Working concentration** | **Lot # (preferred but not required)** | **Persistent ID / URL** |
| --- | --- | --- | --- | --- | --- |
| FAK | Cell Signaling  Technology | 3285 | 1:1000 | - | https://www.cst-c.com.cn/products/primary-antibodies/fak-antibody/3285?site-search-type=Products |
| P-FAK | Cell Signaling Technology | 8556 | 1:1000 | - | https://www.cst-c.com.cn/products/primary-antibodies/phospho-fak-tyr397-d20b1-rabbit-mab/8556?site-search-type=Products |
| PI3K p85 | Cell Signaling Technology | 4257 | 1:1000 | - | https://www.cst-c.com.cn/products/primary-antibodies/pi3-kinase-p85-19h8-rabbit-mab/4257?site-search-type=Products |
| p-PI3K p85 | Cell Signaling  Technology | 17366 | 1:1000 | - | https://www.cst-c.com.cn/products/primary-antibodies/phospho-pi3-kinase-p85-tyr458-p55-tyr199-e3u1h-rabbit-mab/17366?site-search-type=Products |
| AKT | Cell Signaling  Technology | 9272 | 1:1000 | - | https://www.cst-c.com.cn/products/primary-antibodies/akt-antibody/9272?site-search-type=Products |
| P-AKT | Cell Signaling  Technology | 4060 | 1:1000 | - | https://www.cst-c.com.cn/products/primary-antibodies/phospho-akt-ser473-d9e-xp-rabbit-mab/4060?site-search-type=Products |
| Anti-alpha smooth muscle actin | Abcam | Ab7817 | 1:300 | - | https://www.abcam.cn/alpha-smooth-muscle-actin-antibody-1a4-ab7817.html |
| Anti-rabbit IgG, HRP-linked antibody | Cell Signaling  Technology | 7074 | 1:500 | - | https://www.cst-c.com.cn/products/secondary-antibodies/anti-rabbit-igg-hrp-linked-antibody/7074 |
| C1q/Tumor Necrosis Factor-Related Protein 4 | Center for Human Disease Genomics, Peking University | - | Western blot-1:1000;  Immunofluorescence-1:100 | - | Establishment of adipocytokine C1QTNF4 transgenic mouse. DOI：10.3969/j.issn.1671-7856.2014.07.001 |
| Defactinib | Abcam | ab254452 | 10μM | - | https://www.abcam.cn/defactinib-fak-inhibitor-ab254452.html |
| Wortmannin | Cell Signaling  Technology | 9951 | 0.5μM | - | https://www.cst-c.com.cn/products/activators-inhibitors/wortmannin/9951?site-search-type=Products |
| Ki67 | Abcam | ab15580 | 1:50 | - | https://www.abcam.cn/ki67-antibody-ab15580.html |
| CD3 | Abcam | ab16669 | 1:100 | - | https://www.abcam.cn/cd3-antibody-sp7-ab16669.html |
| Caspase-3 | Abcam | ab184787 | 1:200 | - | https://www.abcam.cn/caspase-3-antibody-epr18297-ab184787.html |
| CD31 | Abcam | ab182981 | 1:200 | - | https://www.abcam.cn/cd31-antibody-epr17259-ab182981.html |
| CD68 | Abcam | ab283654 | 1:100 | - | https://www.abcam.cn/cd68-antibody-epr23917-164-ab283654.html |
| Elastin | Abcam | ab9519 | 1:500 | - | https://www.abcam.cn/elastin-antibody-ba-4-ab9519.html |

**DNA/cDNA Clones**

| **Clone Name** | **Sequence** | **Source / Repository** | **Persistent ID / URL** |
| --- | --- | --- | --- |
| C1q/Tumor Necrosis Factor-Related Protein 4 DNA | AF329838 | Homo sapiens | https://www.ncbi.nlm.nih.gov/nuccore/AF329838.1 |

**Cultured Cells**

| **Name** | **Vendor or Source** | **Sex (F, M, or unknown)** | **Persistent ID / URL** |
| --- | --- | --- | --- |
| Rat smooth muscle cell | Peking University Health Science Center | Half F and half M | - |

**Summary of Primer Sequences Used for RT-PCR**

| Gene | Sequence |
| --- | --- |
| C1QTNF4 gene (homo sapiens, NCBI, AF329838) | 5’- AAGCTGATGAAGAACCGCGA-3’  5’- GTAGGCGCCATAGCCATCAT-3’ |
| GAPDH gene: (Mus musculus, NM_001289726.1; Rattus norvegicus (Norway rat) (NM_017008.4) | 5’-CCTTCATTGACCTCAACTACA-3’  5’- GGCAGTGATGGCATGGACTGT-3’ |
| Rattus norvegicus actin alpha 2, smooth muscle (Acta2) (NM_031004) | 5’- GCTATTCAGGCTGTGCTGTC-3’  5’- GGTAGTCGGTGAGATCTCGG-3’ |
| Rattus norvegicus calponin 1 (Cnn1) (NM_031747.2) | 5’- CAGAAATACGACCACCAGAG-3’  5’- ACCTTCTTCACAGATCCCGG-3’ |
| Rattus norvegicus transgelin (Tagln) (NM_031549) | 5’- TTCTTGAAGGCAGCTGAGGA-3’  5’- CCTCTTATGCTCCTGGGCTT-3’ |
